# The FKBP51s Splice Isoform Predicts Unfavorable Prognosis in Patients with Glioblastoma

**DOI:** 10.1158/2767-9764.CRC-24-0083

**Published:** 2024-05-16

**Authors:** Carolina Giordano, Laura Marrone, Simona Romano, Giuseppe Maria Della Pepa, Carlo Maria Donzelli, Martina Tufano, Mario Capasso, Vito Alessandro Lasorsa, Cristina Quintavalle, Giulia Guerri, Matia Martucci, Annamaria Auricchio, Marco Gessi, Evis Sala, Alessandro Olivi, Maria Fiammetta Romano, Simona Gaudino

**Affiliations:** 1 Dipartimento di Diagnostica per Immagini, Radioterapia Oncologica ed Ematologia, Fondazione Policlinico “A. Gemelli” IRCCS, Universitaà Cattolica del Sacro Cuore, Rome, Italy.; 2 Dipartmento di Medicina Molecolare e Biotecnologie Mediche, Università di Napoli, Federico II, Napoli, Italy.; 3 UOC Neurochirurgia, Istituto di Neurochirurgia, Fondazione Policlinico Universitario “A. Gemelli” IRCCS, Università Cattolica S. Cuore, Roma, Italy.; 4 CEINGE Biotecnologie Avanzate, Napoli, Italy.; 5 Istituto di Endocrinologia e Oncologia Sperimentale “Gaetano Salvatore” (IEOS), Consiglio Nazionale delle Ricerche (CNR), Napoli, Italia.; 6 UOS di Neuropatologia, UOC Anatomia Patologica, Fondazione Policlinico “A. Gemelli” IRCCS, Rome, Italy.

## Abstract

The primary treatment for glioblastoma (GBM) is removing the tumor mass as defined by MRI. However, MRI has limited diagnostic and predictive value. Tumor-associated macrophages (TAM) are abundant in GBM tumor microenvironment (TME) and are found in peripheral blood (PB). FKBP51 expression, with its canonical and spliced isoforms, is constitutive in immune cells and aberrant in GBM. Spliced FKBP51s supports M2 polarization. To find an immunologic signature that combined with MRI could advance in diagnosis, we immunophenotyped the macrophages of TME and PB from 37 patients with GBM using FKBP51s and classical M1-M2 markers. We also determined the tumor levels of FKBP51s, PD-L1, and HLA-DR. Tumors expressing FKBP51s showed an increase in various M2 phenotypes and regulatory T cells in PB, indicating immunosuppression. Tumors expressing FKBP51s also activated STAT3 and were associated with reduced survival. Correlative studies with MRI and tumor/macrophages cocultures allowed to interpret TAMs. Tumor volume correlated with M1 infiltration of TME. Cocultures with spheroids produced M1 polarization, suggesting that M1 macrophages may infiltrate alongside cancer stem cells. Cocultures of adherent cells developed the M2 phenotype CD163/FKBP51s expressing pSTAT6, a transcription factor enabling migration and invasion. In patients with recurrences, increased counts of CD163/FKBP51s monocyte/macrophages in PB correlated with callosal infiltration and were accompanied by a concomitant decrease in TME-infiltrating M1 macrophages. PB PD-L1/FKBP51s connoted necrotic tumors. In conclusion, FKBP51s identifies a GBM subtype that significantly impairs the immune system. Moreover, FKBP51s marks PB macrophages associated with MRI features of glioma malignancy that can aid in patient monitoring.

**Significance:**

Our research suggests that by combining imaging with analysis of monocyte/macrophage subsets in patients with GBM, we can enhance our understanding of the disease and assist in its treatment. We discovered a similarity in the macrophage composition between the TME and PB, and through association with imaging, we could interpret macrophages. In addition, we identified a predictive biomarker that drew more attention to immune suppression of patients with GBM.

## Introduction

Glioblastoma (GBM) is the most common and aggressive primary brain tumor in adults, with a poor prognosis owing to its strong tendency to relapse. It exhibits a diffuse invasion pattern; tumor cell infiltration makes complete surgical resection virtually impossible. Despite advances in medical therapy, little progress has been made in improving overall survival (1). Surgery remains the primary therapeutic approach. Wherever possible, the aim of surgery is to achieve massive complete excision of the enhancing solid tumor mass defined by MRI. Indeed, radical surgical resection is associated with improved outcomes in newly diagnosed and recurrent GBM (2).

MRI is a routinely used for diagnosing gliomas, providing a structural overview of the location and detailed anatomy of the tumor. The conventional morphologic MRI includes T1 and T2 weighted images, and gadolinium-enhanced sequences. High-resolution three-dimensional T2* gradient echo sequences, such as susceptibility-weighted imaging (SWI) and diffusion-weighted imaging (DWI), are also included in conventional protocols; the first can differentiate calcification from hemorrhages, and the second one provides information on cell density generating the apparent diffusion coefficient (ADC) map. On morphologic MRI, GBMs typically present as heterogeneous lesions with high cellularity, relevant mass effect, surrounding edema, necrosis, thick rings, or irregular/multinodular contrast enhancement. The combination with low/nonenhancing areas involving the cortex or deep nuclei that cross the contralateral hemisphere via the corpus callosum is particularly suggestive of GBM. Morphologic MRI often fails to identify and quantify infiltrative disease, which may be completely devoid of mass effect and contrast enhancement, resulting in an imprecise definition of the invasive margin (3). Also, it might fail to distinguish contrast enhancement induced by treatment effects in the tumor bed from a residual tumor (4). Advanced imaging techniques, for example, perfusion-weighted imaging and MRI spectroscopy generate a better understanding of tumor biology (5). Used in routine tumor MRI protocols only in larger centers and interpreted by expert neuroradiologists, these techniques support conventional MRI, but do not fully resolve its critical issues.

The tumor microenvironment (TME) has recently been recognized as an essential player and therapeutic target in GBM (6). It contains many myeloid cells and a small proportion of lymphocytes (7–10). The infiltration by bone marrow–derived monocytes with immunosuppressive function increases with glioma grade (8). In contrast, resident microglia participate only marginally in immune suppression (8). Different polarization patterns within M1-M2 opposite phenotypes occur thanks to the plasticity of macrophages (11, 12). A range of macrophage patterns has been recognized in GBM lesions with increasing immunosuppressive capabilities from the marginal area to the center (8). Tumor-associated macrophages (TAM) recirculate in the blood of patients with GBM (13, 14). A previous study conducted by our group on a small population of patients with GBM identified a correlation between certain circulating monocyte subsets marked by FKBP51s, specific MRI features, and treatment response (15).

FKBP51s is encoded by the spliced variant 4 of *FKBP5* gene (16, 17). It plays a relevant role in tumor-related immunosuppression (17), and its expression in macrophages concurs with the acquisition of M2 features (18). FKBP51s offers varying degrees of expression in GBM cancer cell lines and tumors from patients (19) serving as a PD-L1 cochaperone and assisting in protein glycosylation (19). Targeting FKBP51s hampered PD-L1 expression in GBM cells *in vitro* and *in vivo* in an orthotopic mouse model of GBM (19, 20).

In the current study, we use flow cytometry to systematically characterize tumor-infiltrating macrophages and circulating monocyte-macrophage subsets using FKBP51s in addition to conventional immunophenotyping markers. Our study also used flow cytometry to evaluate the expression of markers known to be present in GBMs, such as FKBP51s (19, 20), PD-L1 (14), and HLA-DR (21). Although the cell populations we studied in peripheral blood (PB) may not necessarily originate from TME-infiltrating macrophages (TME-TAM), it is essential to note that they exhibit a phenotype that is not naive and is poorly represented in the blood of healthy individuals (15). Thus, we designated them as “peripheral blood tumor-associated macrophages” (PB-TAM) for simplicity. Correlative studies with MRI parameters allowed us to gain knowledge about TAM phenotypes found in PB and TME. PB-TAMs coexpressing FKBP51s and PD-L1 testified to necrotic tumor while those coexpressing FKBP51s and CD163, found particularly high in recurrences with callosal infiltration, exhibited active STAT6, a transcription factor related to migration and invasion. Moreover, tumor expression of FKBP51s worsened the immunosuppressive TME and was linked to reduced patient survival. This work emphasizes the importance of studying FKBP51s in GBM to better understand its potential utility in clinical practice.

## Materials and Methods

### Patients

From April 2021 to July 2022, we prospectively enrolled 37 consecutive patients diagnosed with GBM (grade IV glioma) according to the latest World Health Organization classification of central nervous system tumors (22). Mean age: 63 (range, 43–78); sex: 25 males and 12 females. Written informed consent was obtained by all patients. Inclusion criteria of the study population were age ≥18 years, suspected GBM diagnosis, clinical indication to perform surgical exeresis, availability of preoperative and postoperative brain MRI with contrast medium, ability to provide written informed consent for study participation. Exclusion criteria of the study population were: patients for whom the histopathologic analysis did not confirm suspected GBM diagnosis, inability to provide written informed consent. The study was conducted in accordance with the principles embodied in the World Medical Association.

### Declaration of Helsinki

The study was approved by the Institutional Review Board of Fondazione Policlinico Universitario Gemelli (FPG; Prot ID 3751, 1/07/2021). All patients were treated at the Neurosurgery Division of FPG and managed according to guidelines (23). All patients received corticosteroids for 1 week before surgery. All recurrences have been free of treatment (Stupp) for at least 2 months. The clinical information and study results were handled only by authorized personnel. Patient identity was kept confidential in compliance with the patient's rights. Of the 37 patients with GBM, 27 had a primary tumor and 10 were hospitalized and underwent surgery for suspected recurrence (confirmed by pathology). Thirty-three patients underwent complete immunophenotyping of the TME and PB, as described in the *Flow cytometry analysis* paragraph. In 3 patients, partial data were obtained on TME due to the scarcity of tumor material. The PB of 1 patient was not available. Fifteen patients underwent presurgical MRI at the FPG Radiology Unit, while 22 patients at other centers made it available for reading. The MRI scans of three patients performed in other centers did not include the SWI sequence. Patient details are summarized in Supplementary Table S1. A flow chart of study population is shown in Supplementary Fig. S1.

### Imaging

All examinations were performed using a 1.5 T MRI scanner with an eight-channel head coil. All patients underwent turbo spin-echo (TSE) T1-weighted imaging (T1WI) and T2WI, fluid-attenuated inversion recovery (FLAIR), DWI, SWI, and contrast-enhanced T1 TSE WI.

Qualitative analysis was used to evaluate the following morphologic features: tumor location, macroscopic hemorrhage, ependymal enhancement (EE), corpus callosum infiltration (CCI), and midline shift (MS). Quantitative analysis included the measurement of tumor volumes (TV), including volume of necrosis, intratumoral susceptibility signal scores (ITSS), perilesional infiltrative and vasogenic edema (VE), and ADC. The tumor and necrotic area volumes were obtained with a dedicated workstation by analyzing a freehand-drawn region of interest (ROI) with a semiautomatic system. TV was calculated for all primary lesions except for two cases of GBMs lacking a “compact” component and showing exclusively infiltrating characteristics. TV, necrosis score (NS), ITSS, and vasogenic edema were not considered in the recurrences due to the paucity and irregularity of lesions that the postradiotherapy alterations could somehow mislead.

TV was defined on a T1-weighted contrast-enhanced image, and necrosis volume was defined as the central, nonenhancing tumor part (24). A simple division of their volumes yielded the relative extent of necrosis within the tumor (necrosis/tumor ratio = N/T). NS was assigned on the basis of the percentage of intratumoral necrosis (score 1, <5%; score 2, between 5% and 20%; score 3, >20%; ref. 24). ITSS was measured on SWI images and scored according to Gaudino and colleagues (ref. 25; as follows:0 = absence of ITSS, 1 = presence of 1–10 ITSS, 2 = presence of ≥11 ITSS). Measurement of perilesional infiltrative and VE was automatically segmented on T2 and FLAIR images and scored as 0: less than half, or 1: more than half of the entire abnormality (26). ADC values were measured on DWI sequence maps obtained by drawing an ROI of approximately 20 mm2 in correspondence of the enhancing portion of the tumor and peritumoral region. In the preoperative MRI of the 10 recurrent tumors, only EE, CCI, MS, and ADC values were considered because of the paucity of pathologic tissue altered by postradiotherapy remodeling. The MRI features of the study population are summarized in Supplementary Table S1.

### Peripheral Blood Mononuclear Cells Isolation and Tumor Specimens’ Dissection

At the time of surgery, 5  µL of blood was collected from each patient in a sterile K3EDTA vacutainer tube. Peripheral blood mononuclear cells (PBMC) were separated by differential centrifugation using a Ficoll-Hypaque density gradient (Histopaque-1077; Sigma-Aldrich), washed, and resuspended in RPMI1640 medium (Corning) supplemented with 10% heat-inactivated FBS (Corning), 200 mmol/L glutamine (Lonza), and 100 U/mL penicillin-streptomycin (Lonza). After immersion in ice-cold 10% FBS RPMI1640 medium, the GBM tissue was mechanically dissociated as described previously (27). For the study of the TME, dissociated cells, containing both tumor and immune infiltrates, were subjected to immunofluorescence as described in *Flow cytometry analysis* paragraph.

### Cell Culture and Transfection

All cell lines were maintained at 37°C in a 5% CO_2_ humidified atmosphere. GB138 primary culture was established from acutely resected human GBM originated from a woman and was kind gift from Prof. Rogister (Laboratory of Developmental Neurobiology, GIGA-Neurosciences, University of Liege, Liege, Belgium; ref. 28). GB83 primary culture was established from human tissue of mesenchymal GBM subtype derived from a 52-year-old male patient and kindly gifted by Dr. Ricci-Vitiani (Department of Oncology and Molecular Medicine, Istituto Superiore di Sanità, Rome, Italy; ref. 29). No authentication was performed for the above-described cell lines. U87 is a cell line with epithelial morphology that was isolated from a male patient with GBM and was purchased and authenticated by short tandem repeat profiling by the ATCC. D54 cells originate from a 53-year-old male patient with GBM; human monocytic THP-1 cells derive from acute monoblastic/monocytic leukemia of a male child of 1 year. These latter were obtained and authenticated from the CEINGE cell bank facility (https://www.ceinge.unina.it/en/cell-cultures). Each cell line was tested for *Mycoplasma* after every thawing using a PCR-based method suitable for the detection of 11 mollicutes and capable of detecting all *Mycoplasma* species as indicated by Molla Kazemiha and colleagues (30). After thawing, the cells were used in a range of passage numbers from the 4th to the 10th–12th to keep safe the cell line identity. GB138 and U87 cells were cultured in DMEM (Corning), whereas GB83 and D54 cells were cultured in DMEM/Hams F-12 v/v (Corning). Human monocytic THP-1 cells (CEINGE Cell Bank) were cultured in RPMI1640 (Corning). All media were supplemented with 10% heat-inactivated FBS (Corning), 200 mmol/L glutamine (Lonza), and 100 U/mL penicillin-streptomycin (Lonza). For overexpression and silencing experiments, cells were seeded in 6-multiwell plates at a density of 4 × 10^5^ per well to obtain 60%–70% confluency. After 24 hours, cells were transfected with the Metafectene Transfection System (Biontex) in accordance with the manufacturer's recommendations; 3 µg of the plasmid of interest (Supplementary Materials and Methods) was transfected per well. Transient knockdown of FKBP51s was performed using a mix of three custom siRNAs produced by Qiagen at a final concentration of 50 nmol/L, using No Sense RNA as a control (Supplementary Materials and Methods). Cells were harvested 36 hours after transfection.

### Generation of Spheroids

The spheroids were generated as described by Garnier and colleagues (31). Briefly, GB83 or GB138 cell lines were plated at a density of 1,000 cells/mL and grown in serum-free medium supplemented with 20 ng/mL of recombinant EGF and 10 ng/mL FGF-2 (Sigma-Aldrich) and allowed to grow in low-attachment condition for 7–10 days. Cancer stemness was evaluated in dissociated GBM spheres by assessing the expression of stemness markers (CD133, EPHA2, NANOG, OCT 3/4, SNAIL, SOX2, and ZEB1) using qPCR (see *qPCR* paragraph).

### Cocultures of GBM Cells and THP-1

THP-1 monocytes (1 × 10^5^cells/mL) were seeded into a 6-well plate and differentiated into M0 macrophages by a 36-hour incubation with 100 ng/mL phorbol 12-myristate 13-acetate (Sigma-Aldrich; ref. 32). Next, the cells were washed three times with PBS and cultured for 36 hours in RPMI medium. GB138 and GB83 differentiated cells or spheroids (1 × 10^5^ cells/mL) were seeded with THP1-derived macrophages and cocultured in DMEM for 24 hours. Spheroids in suspension were collected and separated by centrifugation of supernatants. Adherent GBM cells and THP-1–derived macrophages were harvested by trypsinization and distinguished by CD45 antigen expression by flow cytometry (*Flow cytometry analysis*). In each experiment, THP-1-derived macrophages and adherent and spheroid GB138 and GB83 cells were maintained in monocultures as controls.

### Flow Cytometry Analysis

The BD-Pharmigen Fc block (2.5 µg/10^6^ cells) was used to minimize the nonspecific binding of immunoglobulins to Fc receptors before immunostaining (18). PBMCs were resuspended at a concentration of 2 × 10^6^/mL. A total of 5–10 µL (in accordance with manufacturer's instructions) of mouse mAb (Supplementary Materials and Methods) were added to 50 µL of PBMC suspension, as described previously (18), and incubated for 15 minutes in the dark at room temperature (20°C–25°C). Next, 200 µL of a fixation/permeabilization buffer (BD-Pharmingen Cytofix/Cytoperm Kit) for intracytoplasmic staining and Transcription Factor Staining Buffer Set (eBioscience, Thermo Fisher Scientific) for nuclear staining were added to each tube and incubated for 20 minutes in the dark at 4°C. The antibodies used for intracellular staining are listed in Supplementary Materials and Methods. For each staining, an Ig isotype-conjugated antibody was used as a control for nonspecific binding. Samples were acquired using a BD Accuri C6 Cytometer (Becton, Dickinson and Company BD) and analyzed using FlowJo or C6 Accurì software.

### Multiplex Bead-based Flow Cytometry

Serum samples were subjected to the human MACSplex cytokine 12-kit (Miltenyi Biotec GmbH) to quantify the concentrations of GMCSF, IFNα, IFNγ, lL2, IL4, IL5, IL6, IL9, IL10, IL12p70, IL17A, and TNFα according to the manufacturer's instructions (see also Supplementary Materials and Methods for details).

### qPCR

Total RNA was extracted using TRIzol (Sigma-Aldrich) according to the manufacturer's instructions. A total of 1 µg of each RNA sample was used for cDNA synthesis using iScript Reverse Transcription (Bio-Rad). Gene expression was quantified by qPCR using SsoAdvanced SYBR Green Supermix (Bio-Rad) and Bio-Rad CFX96 Real-Time PCR detection system according to the manufacturer's instructions. Specific qPCR primers for the relative quantification of the transcripts were employed using coamplified SDHA, RPS18, or BACT as an internal control for normalization. The absolute quantification of transcripts was calculated using the 2ˆ−DeltaCt method (DeltaCt = Ct of the target gene − Ct of the housekeeping gene) to represent the expression differences between different patients. Relative expression 2^−ΔCt^ ± SD and the mean fold change = 2^−(average ΔΔCt)^ ± SD were calculated using the mean difference in ΔCt between the genes and internal control. ΔCt was calculated using the differences in the mean Ct values between the genes and internal control. Oligo sequences are reported in Supplementary Materials and Methods.

### Immunoblot

Whole-cell lysates were prepared as described previously (17) and assayed by immunoblotting. Briefly, samples were denatured for 5 minutes at 95°C, then loaded in 8%/10% T SDS-PAGE, and transferred into a methanol-activated polyvinylidene difluoride membrane (Immobilon-P; Sigma-Aldrich). The membranes were incubated with the primary antibody at 4°C overnight. Primary antibodies Supplementary Materials and Methods) were diluted as follows: anti-phospho-STAT3, anti-FKBP51, and anti-FKBP51s were used at a dilution of 1:2,000. Anti-phospho-STAT6, anti-STAT3, and anti-STAT6 antibodies were used at dilution 1:500. Anti-β-Actin, anti-Vinculin, anti-G3PDH and anti-γ-tubulin were used at the dilution of 1:5,000. After washes, membranes were incubated with secondary antibodies for 1 hour, at room temperature. Anti-mouse and anti-rabbit secondary antibodies horseradish peroxidase–conjugated (ImmunoReagents) were diluted at 1:5,000. The protein bands were visualized with a chemiluminescence detection system (Western Blotting Luminol Reagent, Santa Cruz Biotechnology). Quantification of bands was obtained by densitometry analysis using ImageJ 1.42q for Macintosh, see details in Supplementary Materials and Methods.

### Statistical Analysis

The subjects were divided into two groups using Ward clustering method based on the variable parameters measured in the tumor specimens. We used the Mann–Whitney, the *χ*^2^, Fisher, and Student *t* tests, when appropriate, to assess the differences between the two groups. The Welch correction was applied in case of two sample means significantly different, but variances and/or the sample size were unequal. To plot the heat maps, the data were scaled to row-wise Z-scores. ANOVA and Pearson correlation tests were used for multiple comparisons and correlative studies. For the Kaplan–Meier survival estimate, patients were divided according to the median FKBP51s-tumor expression value [197 mean fluorescence intensity (MFI), range 0/4134 MFI]. Statistical significance was set at *P* ≤ 0.05. Analyses were performed using the R environment for statistical computing (v4.1.0) and GraphPad 7.0a for Macintosh. Cox regression served to multivariate analysis.

### Data Availability

The data generated in this study are included in this article and its Supplementary Data.

## Results

### Tumor-infiltrating Macrophages of GBM Tumors Distinguish Two Groups of Patients

**FIGURE 1:**
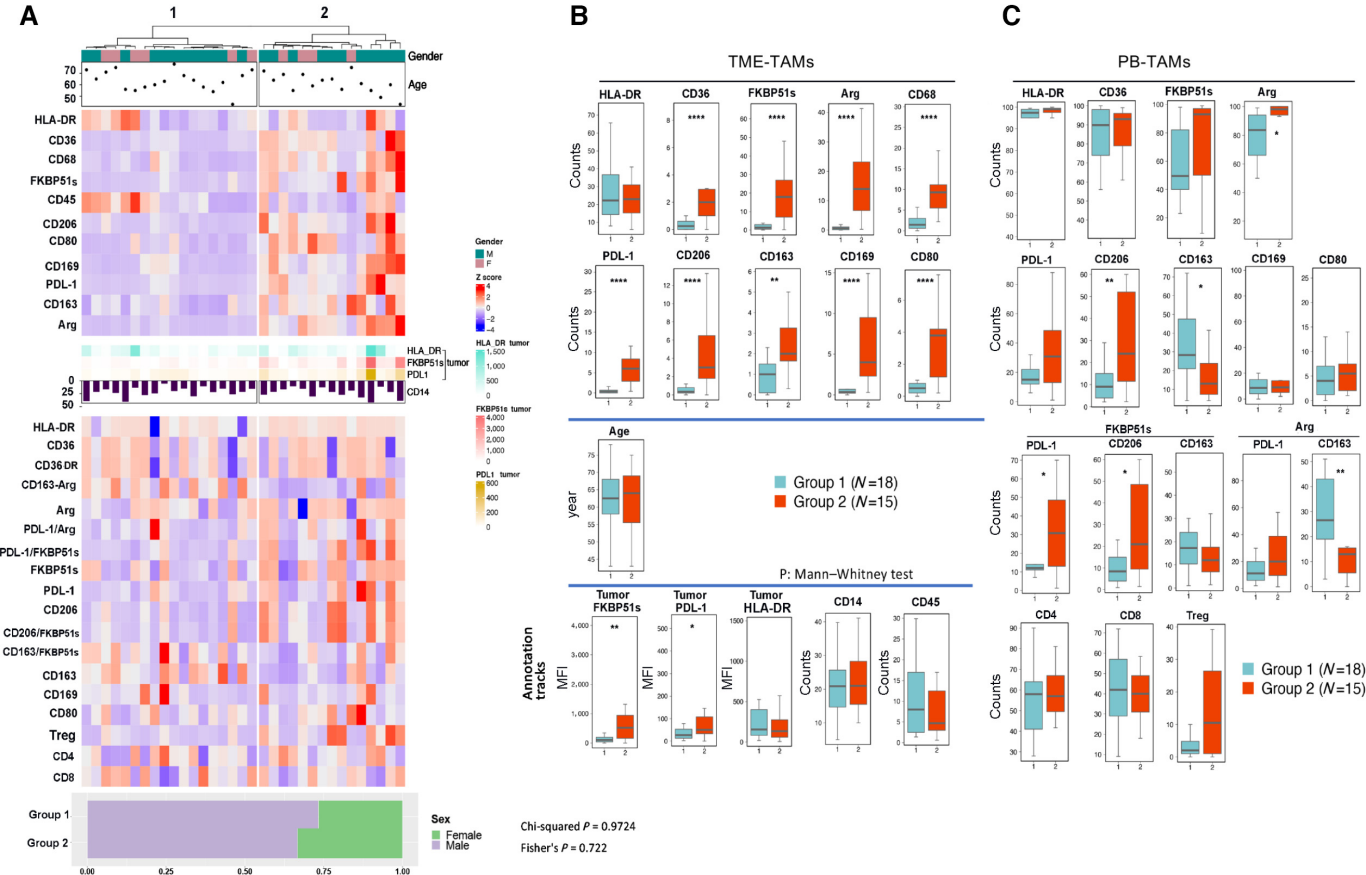
GBM TME analysis clusters two groups that differ in TAM composition. **A,** The subjects were divided into two groups using the Ward hierarchical clustering method based on the variable parameters measured in the TME (top heat map). The data from PB (bottom heat map) were not used for clustering, they followed the separation of TME data. Age, gender, HLA-DR tumor, FKBP51s tumor, PD-L1 tumor, and % of total infiltrating CD14 are reported as annotations of the heat map. **B,** Values of TME immunophenotype measured for each cluster are reported as box plots. **C,** Values of PB immunophenotype are reported as box plots. The *P* values of statistical difference tests are shown. *, *P* < 0.05; **, *P* < 0.01; ***, *P* < 0.001; ****, *P* < 0.0001.

Clustering of TME data (Fig. 1A, top) identified two groups that differed significantly in the frequency of M2 macrophages (CD206, CD163, PD-L1, CD36, CD169, Arginase, FKBP51s) that were increased in group 2. An increased proportion of CD68 and CD80 macrophages was also observed in group 2 (Fig. 1B). No difference was recorded between CD45 cells and CD14 macrophages infiltrating the tumor (Fig. 1B). The frequency (Fig. 1B) and the brightness (Supplementary Fig. S2) of HLA-DR macrophages was similar between the two groups (Fig. 1B). FKBP51s, PD-L1, and HLA-DR expressions were also measured in tumor cells and included as annotation tracks (Fig. 1A and B). Tumors clustered in group 2 expressed FKBP51s and PD-L1 at higher levels, in comparison with group 1 tumors. For details of immunophenotyping of tumor samples, see Supplementary Materials and Methods. PB immunophenotyping was performed using the same M1 and M2 markers employed to analyze the TME biparametrically with FKBP51s and Arg, and analysis was carried out as described previously (15). The number of CD3 lymphocytes (CD4, CD8, and regulatory T cells/Treg) was measured. The data (Fig. 1A, bottom) are represented as box plots in Fig. 1C. A general increase in CD14/Arg cells was observed in group 2, along with the counts of PD-L1/FKBP51s, CD206, and CD206/FKBP51s macrophages. CD163 macrophages showed an opposite trend, as their counts (and those of CD163/Arg) were increased in group 1. No difference was found in CD163/FKBP51s between the two groups. Flow cytometry results are provided as Supplementary, Immunophenotyping values.

### Tumor Expression of FKBP51s is Associated with Immunosuppressive TME and Systemic Tolerogenic Immune Condition

**FIGURE 2:**
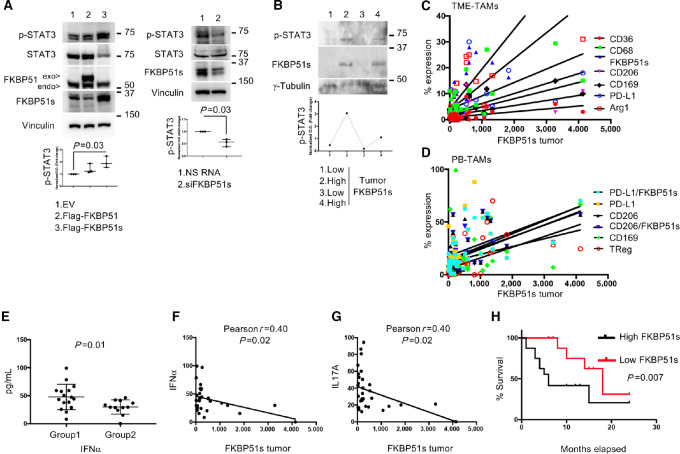
FKBP5 splicing in GBM tumors sustains STAT3 activation and is accompanied by systemic tolerogenic immune condition. **A,** Left, IB of GB138 cell transfected with Flag-FKBP51, Flag-FKBP51s or correspondent EV as control. The spliced FKBP51s, but not the canonical FKBP51, increased pSTAT3 levels (*N* = 3). Right, IB of GB138 cell transfected with siFKBP51s or a nonsilencing RNA (NS) as control. Compared with control (NS) cells, siFKBP51s treated cells showed impaired levels of pSTAT3 (*N* = 3). **B,** Immunoblot of pSTAT3 expression 4 GBMs, samples 2 and 4 show that pSTAT3 corresponded to FKBP51s expression. Linear regression of tumor-FKBP51s with TME-TAMs (**C**) and PB-TAMs (**D**). The Pearson correlation coefficient test calculated a positive correlation with the indicated TAMs (see Supplementary Table S2). **E,** IFNα levels in clusters 1 and 2. **F** and **G,** Linear regression of tumor-FKBP51s and IFNα or IL17A. **H,** Kaplan–Meier curves for OS rates, compared between FKBP51s^high^ and FKBP51s^low^ patients. The two groups were divided according to the median value of tumor FKBP51s expression. EV = empty vector; IB = immunoblot.

Because the group 2 was characterized by tumor-FKBP51s expression significantly higher than that of group 1 (Fig. 1B, bottom), we investigated whether FKBP51s regulated STAT3 activation, a transcription factor that sustains GBM-induced immunosuppression (33). To this end, we used the GB138 cell line established from a patient tumor (28) and modulated the expression of FKBP51s. As shown in Fig. 2A (left), overexpression of FKBP51s, but not canonical FKBP51, upregulated the pSTAT3 levels. Conversely, depletion of FKBP51s reduced such levels (Fig. 2A, right). Immunoblots of other three GBM cell lines confirm these results (Supplementary Fig. S3). The association between tumor FKBP51s expression and pSTAT3 levels was observed in four GBM samples from patients (Fig. 2B). Correlative analyses of tumor FKBP51s-expression with TME-TAMs calculated an association with several M2 phenotypes, specifically: CD206 (Pearson *r* = 0.5; *P* = 0.001), PD-L1 (Pearson *r* = 0.5; *P* = 0.001), FKBP51s (Pearson *r* = 0.8; *P* < 0.0001), CD36 (Pearson *r* = 0.5; *P* = 0.002), CD169 (Pearson *r* = 0.7; *P* < 0.0001), and Arg (Pearson *r* = 0.7; *P* < 0.0001; Fig. 2C), with the microglia marker CD68 (Pearson *r* = 0.6; *P* < 0.0001) and, as expected (16, 19) with tumor-PD-L1 expression (Pearson *r* = 0.9; *P* < 0.0001). No correlation was observed with other TME markers (Supplementary Table S2). In addition, Pearson correlation coefficient test revealed an association between tumor-FKBP51s expression and various PB-TAMs, specifically: CD169 (*r* = 0.32; *P* = 0.05), CD206 (*r* = 0.52; *P* = 0.001), CD206/FKBP51s (*r* = 0.55; *P* = 0.0005), PD-L1 (*r* = 0.48; *P* = 0.03), PD-L1/FKBP51s (*r* = 0.6; *P* = 0.0001), and Tregs (*r* = 0.57; *P* = 0.0003; Fig. 2D). No correlation was observed with other immunophenotypes (Supplementary Table S3). Flow cytometry results of FKBP51s expression were confirmed by immunoblotting of seven GBM protein extracts (Supplementary Fig. S4). Examining cytokine levels in patient sera revealed a significant increase in IFNα in group 1 versus group 2 (Fig. 2E). Although such an increase appeared negligible when correction for multiple comparisons was applied (Supplementary Fig. S5), linear regression analysis revealed that IFNα (Fig. 2F) and IL17A negatively correlated with tumor FKBP51s (Fig. 2G). Tumor FKBP51s connoted patients with GBM with reduced survival (Fig. 2H). Multivariate analysis revealed that the association always remains significant (*P* = 0.02), adding possible confounders (Table 1). The finding that low levels of proinflammatory cytokines corresponded to increased M2 and Treg counts supports an anti-inflammatory and immunosuppressive condition in FKBP51s tumors. As several MRI characteristics of tumor aggressiveness were independent of this biomarker (Supplementary Table S4), it is reasonable that a pronounced immunosuppression could sustain the unfavorable outcome of FKBP51s tumors.

**TABLE 1:** Cox regression analysis for overall survival with multiple variables

			95.0% CI for Exp(B)	
Factors	*P* value	Exp (B)	Lower	Upper
Tumor FKBP51s (High, Low)	0.026	30.347	1.501	613.692
Age (y)	0.070	1.198	0.986	1.456
Sex (M/F)	0.102	10.002	0.631	158.601
Corpus callosum infiltration (no, yes)	0.754	0.739	0.112	4.899
Tumor volume (cm^3^)	0.451	0.989	0.960	1.019
Resection (total, partial)	0.098	0.033	0.001	1.886
Midline shift (no, yes)	0.629	0.575	0.061	5.414
ADC value 10^−6^ mm^2^/second	0.745	0.999	0.996	1.003

### CD163/FKBP51s PB-TAMs are a Common Feature of GBM Tumors

**FIGURE 3:**
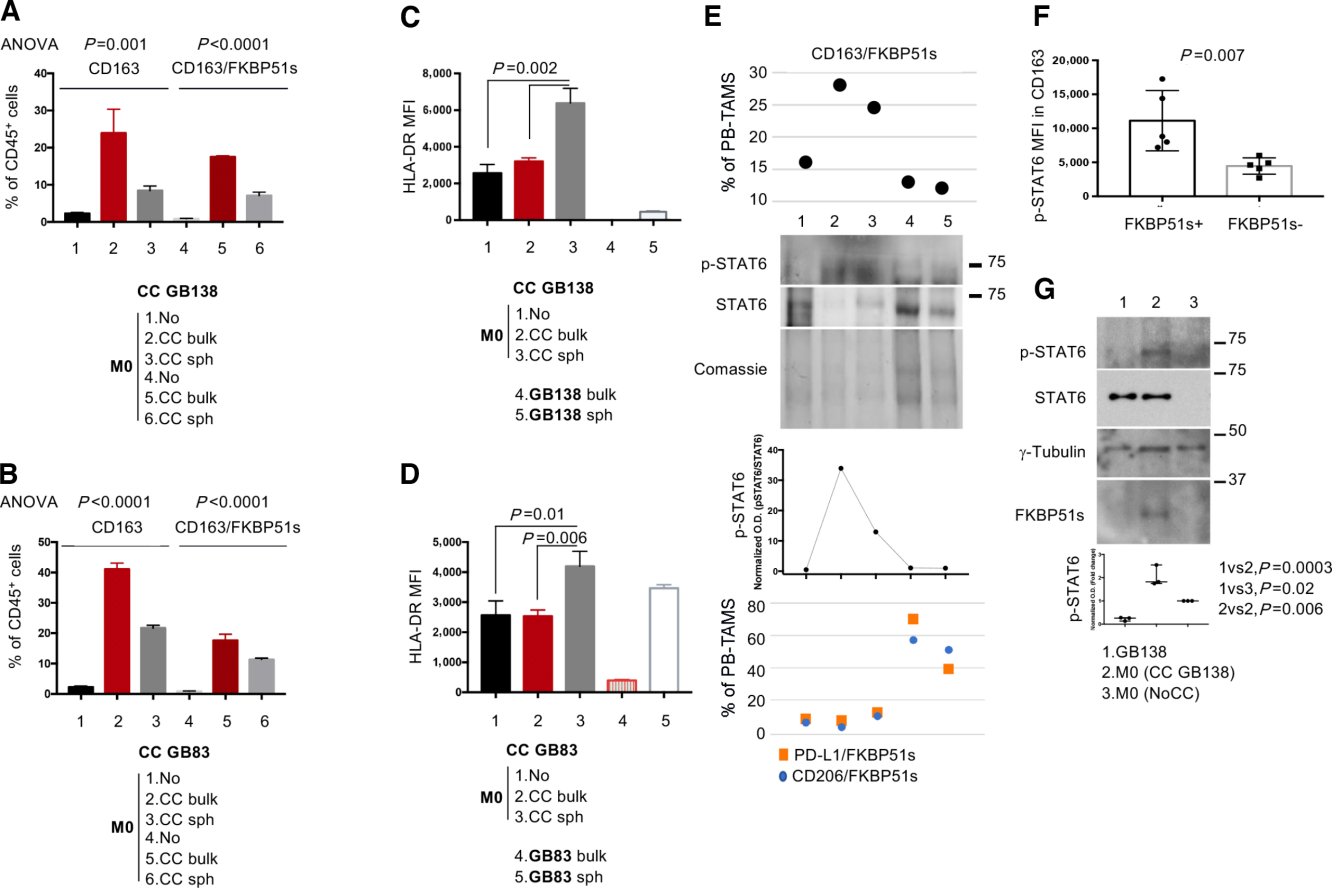
Effect of the tumor on macrophage polarization. Cocultures of M0 cells with GB138 (**A**) and GB83 (**B**), either adherent cell or spheroid. Expression of CD163 on macrophages was upregulated in cocultures with adherent cells (red column) more than spheroids (gray column). Part of CD163 macrophages expressed FKBP51s (*N* = 3). Cocultures of M0 cells with GB138 (**C**) and GB83 (**D**), either adherent cell or spheroid. Expression of HLA-DR (MFI) on macrophages was upregulated in cocultures with spheroids (gray column). HLA-DR's expression was also registered on tumor cells, particularly spheroids (*N* = 3). **E,** Immunoblot of pSTAT6 expression in PBMCs of 5 patients with GBM. Samples 2 and 3 show pSTAT6. Corresponding values of CD163/FKBP51s (top) and PD-L1/FKBP51s or CD206/FKBP51s (bottom) PB-TAMs are also shown. **F,** Graphic representation of pSTAT6 levels (MFI) in CD163 macrophages from cocultures (*N* = 5). Coexpression of FKBP51s in CD163 significantly increased pSTAT6 (see Supplementary Fig. S5F and S5G). **G,** Immunoblot of pSTAT-6 levels in cocultures. Tumor cell–macrophage interaction induced STAT6 activation (*N* = 3).

A previous study found that CD163/FKBP51s cells in the PB increased following incomplete tumor resection (15), suggesting that tumor persistence associated with disruption of the blood-brain barrier promotes the release of these TAMs into circulation. As GBM immune edits infiltrating myeloid cells (7) and given that CD163/FKBP51s are equally distributed in the two clusters (Fig. 1C), we investigated the role of the tumor in generating this macrophage phenotype. To this end, we cocultured M0 macrophages and tumor cells using patient-derived cell lines GB138 (28) and GB83 (29). Cocultures were performed with M0+adherent cells or M0+spheroids. Supplementary Figure S6 shows enriched expression of stemness genes in spheroid-forming cells, in comparison with cells grown in adhesion. The cells were subjected to quadruple staining using CD163, FKBP51s, HLA-DR, and CD45 (for gating, see Supplementary Fig. S7A). Our results show that CD163/FKBP51s macrophages were induced by the interaction of M0 with adherent tumor cells (bulk tumor) more than spheroids (stem cell component; Fig. 3A and B; Supplementary Fig. S7B). Interestingly, we found that HLA-DR was significantly upregulated in M0 macrophages cocultured with spheroids (*P* = 0.002 and *P* = 0.01 for cocultures with GB138 and GB83 spheroids, respectively; Fig. 3C and D; Supplementary Fig. S7C). Tumor cells, particularly spheroids, were also stained for HLA-DR (Fig. 3C and D, white histograms). These results suggest that bulk tumor and cancer stem cells have opposite effects on macrophage polarization, inducing M2 and M1 phenotypes, respectively. This hypothesis is supported by the quantification of transcript levels of *IL4* and *IL10*, which are crucial factors in alternative macrophage polarization (12, 32), in adherent tumor cells and spheroids (Supplementary Fig. S7D and S7E), showing a significant difference in expression in favor of bulk tumor (GB138 spheroid vs. bulk: *P* = 0.017 and *P* = 0.009 for *IL10* and *IL4*; GB83 spheroid vs. bulk: *P* = 0.001 and *P* = 0.0029 for *IL10* and *IL4*). Among heterogeneous M2 macrophages, a subgroup, identified by pSTAT6 expression, exhibits the most protumoral properties (12) and migration ability (34, 35). Protein extracts from 5 GBM patients’ PBMCs showed that expression of pSTAT6 (Fig. 3E lanes 2 and 3) was associated with increased counts of CD163/FKBP51s PB-TAMs (Fig. 3E, top) but not PD-L1/FKBP51s or CD206/FKBP51s (Fig. 3E, bottom). We then measured pSTAT6 expression in macrophages cocultured with GBM cells using flow cytometry. Cocultures were stained with CD45, CD163, FKBP51s, and pSTAT6. After gating CD45 cells, a second gate was placed on CD163/FKBP51s^+^ or CD163/FKBP51s^−^ cells (Supplementary Fig. S7F). Measurement of pSTAT6 (Fig. 3F; Supplementary Fig. S7G) showed that FKBP51s remarkably increased its level. Activation of pSTAT6 in the cocultures was confirmed by immunoblotting (Fig. 3G).

### Immunologic Markers as Indicators of Imaging Profile

To gain more knowledge about various TAMs measured in patients with GBM, we carried out a study to identify a possible association between TAMs and the MRI parameters used to evaluate the malignancy characteristics of gliomas. To this end, we performed a correlative analysis of TAMs with MRI profiles, which also involved examining immunologic markers expressed by the tumor. As radiologic phenotypes are often influenced by treatment (36), primary and recurrent tumors were analyzed separately. Supplementary Figure S8 shows a comparison between the flow cytometry data of TME and PB from primary tumors and recurrences. The only difference observed was in HLA-DR and CD206 TME-TAMs, which were higher in primary tumors than in recurrences. No significant differences were registered between PB-TAMs counts. Given the availability of MRI data, CCI, ADC, MS, and EE were analyzed in either primary or recurrent tumors (Supplementary Figs. S9–S12), whereas TV, NS, ITSS, and VE were analyzed in primary tumors only (Supplementary Figs. S13–S16).

Significant associations were found between immunophenotyping values from TME or PB with CCI (Supplementary Fig. S9), ADC (Supplementary Fig. S10), MS (Supplementary Fig. S11), TV (Supplementary Fig. S13), and NS (Supplementary Fig. S14).

**FIGURE 4:**
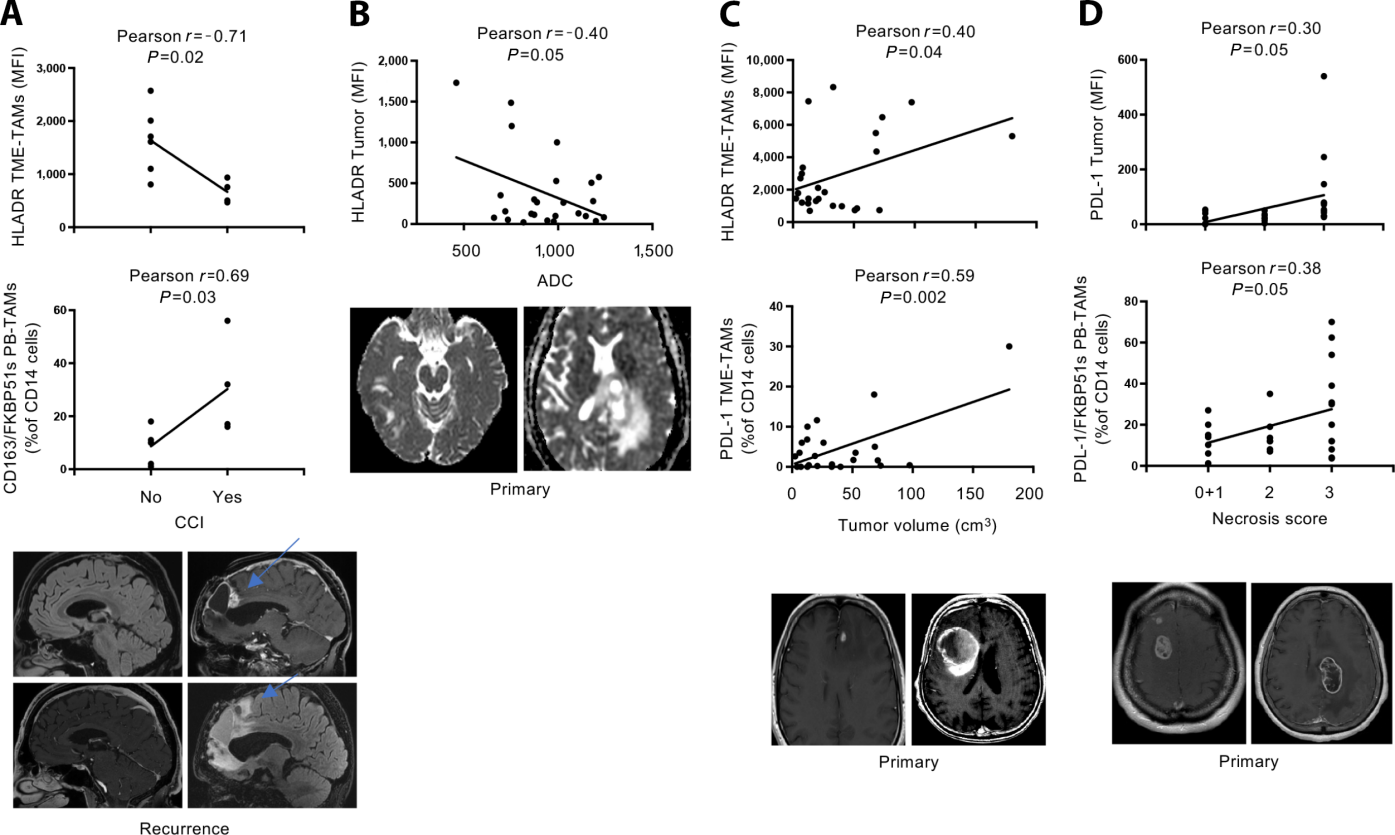
Immunologic markers as indicators of imaging profiles. **A,** Linear regression of MFI values of HLA-DR TME-TAMs and the counts of CD163/FKBP51s PB-TAMs versus CCI (upper images). T2 FLAIR and (lower images) T1 with gadolinium on the sagittal plane, passing through the CC. No alteration of the CC signal in the left images. The right images show the infiltration of the anterior trunk of the CC (arrows). **B,** Linear regression of tumor HLA-DR expression and ADC. Representative ADC maps of left hemispheric GBMs. The signal of the solid tumor component appears hyperintense in a GBM of the frontoparietal lobe in the left hemisphere (right, high ADC value), low intense signal in a GBM of the temporal lobe of the right hemisphere (left, low ADC value). **C,** Correlation of TV versus HLA-DR brightness of TME-TAMs or versus counts of PD-L1 TME-TAMs. Axial T1w images after gadolinium show on the left a GBM with a small focal area of enhancement in the left frontal lobe (small TV). In contrast, the image on the right shows a GBM with an extensive enhancement area in the right frontal lobe (large TV). **D,** Correlation of NS with tumor PD-L1 expression and counts of PD-L1/FKBP51s PB-TAMs. T1w axial images after gadolinium: (left) frontal GBM in the right hemisphere shows vivid enhancement, which spares only small necrotic areas (NS: 1); (right), GBM in the frontoparietal lobe of the left hemisphere shows predominantly peripheral gadolinium enhancement with a large central necrotic component (NS: 3).

Specifically, in recurrences with CCI, the brightness of infiltrating HLA-DR TAMs was significantly decreased, compared with recurrences without CCI (*P* = 0.02), and a concurrent increase in CD163/FKBP51s PB-TAMs (*P* = 0.03) was registered (Supplementary Fig. S9). Pearson correlation test confirmed a negative correlation of CCI with HLA-DR brightness of TME-TAMs (*r* = −0.71; *P* = 0.02) and CD163/FKBP51s PB-TAMs (*r* = 0.69; *P* = 0.03). Figure 4A shows representative MRI of recurrences without or with CCI.

In primary tumors, cell density generating the ADC map was related to tumor HLA-DR expression (Pearson *r* = −0.40; *P* = 0.05; Fig. 4B; Supplementary Fig. S10).

A linear correlation subsisted between TV and either PD-L1 (Pearson *r* = 0.63; *P* = 0.001) and HLA-DR^bright^ TME-TAMs (Pearson *r* = 0.40; *P* = 0.01; Fig. 4C). Also, tumors with MS showed an increased infiltration with HLA-DR^bright^ TME-TAMs (Supplementary Fig. S11). TV and midline shift were MRI-associated features (Supplementary Table S5).

Regarding tumor necrosis, ANOVA analysis revealed variations in FKBP51s TME-TAMs and CD163 and CD163/FKBP51s PB-TAMs in three patient groups categorized according to the NS (0+1, 2,3; Supplementary Fig. S14). A linear association of NS with tumor PD-L1 expression (Pearson *r* = 0.30; *P* = 0.03) and PD-L1/FKBP51s PB TAMs (Pearson *r* = 0.38; 0.05; Fig 4D; Supplementary Table S6) suggested that PD-L1–expressing tumors are more prone to necrosis. An association was found between NS with ITSS score (Supplementary Table S7). The intratumoral areas of low signal on susceptibility images rely on multiple factors additional to necrosis, including intratumoral microhemorrhage, calcification, and neovascularization. We did not find any correlation between ITSS and TAMs (Supplementary Fig. S15; Supplementary Table S8).

## Discussion

Our study characterized TAMs in GBM using FKBP51s along with classical M1 and M2 macrophage markers. Through TAM correlation with MRI profiles, we gained a deeper understanding of the significance of the different macrophage phenotypes in GBM. At the same time, our examination of tumor cells revealed that the spliced isoform of the FKBP5 gene can also serve as a prognostic marker for GBM.

We identified two distinct TME groups that were sorted on the basis of the clustering of different TME-TAM phenotypes. TME of the second cluster was found to be rich in M2 macrophages, and tumors with high FKBP51s expression were clustered in this second group. In PB, a range of M2 phenotypes, a Th17/Treg balance skewed toward Tregs, and reduced levels of type I IFN were all associated with GBM tumors expressing high FKBP51s levels. Such tumors exhibited STAT3 activation and were associated with poor survival. On the basis of the data that several MRI characteristics of tumor aggressiveness were found to be independent of this biomarker, it is reasonable to conclude that a pronounced immunosuppression could be a key factor contributing to the unfavorable outcome of FKBP51s tumors.

Coculture assays have revealed that GBM cells grown in adherence facilitate the development of CD163/FKBP51s macrophages, which exhibited active STAT6 levels, a transcription factor enabling migration and invasion properties of macrophages (34, 35). This finding has led to speculation about the possibility that development of these macrophages could occur in the brain helping infiltration and invasion in the brain parenchima of malignant glioma cells. The evidence shows that in cases of recurrences with callosal infiltration, there is a significant increase in CD163/FKBP51s PB-TAMs as compared with the cases without CCI. On the other hand, spheroids tend to promote M1 polarization, which is indicated by the intense HLA-DR expression (37, 38). This finding suggests that M1 macrophages may infiltrate alongside cancer stem cells. The drastic decrease in infiltrating M1 macrophages accompanying the increase in CD163/FKBP51s PB-TAMs raises the hypothesis of the relocation of cancer stem cells to other brain areas. This hypothesis deserves in-depth investigation to gain valuable insights into the mechanisms of cancer progression in the brain.

A vigorous HLA-DR intensity in tumor-infiltrating macrophages positively correlated with TV. Zhou and colleagues found a correlation between M1 macrophage infiltration and poor survival outcomes and therapy resistance of GBMs (39). Results of coculture with spheroids suggest that high M1 infiltration could reflect the content of glioma stem cells that replenish the tumor.

Our study revealed that GBMs with large necrotic areas express PD-L1 and have increased PD-L1/FKBP51s PB-TAMs, consistent with a previous study (15). With its precise detection capabilities, MRI can accurately identify necrotic regions that suggest a grade IV glioma (18) and poor prognosis (40). This TAM subset can serve as a valuable marker to support MRI diagnosis, thereby improving the accuracy and efficacy of glioma diagnosis.

HLA-DR belongs to MHC class II antigens that, differently from MHC I, are not ubiquitously expressed by tissue cells but are involved in antigen presentation to effector lymphocytes and are expressed in limited subsets of immune cells, including macrophages and activated CD4 T cells. Diao and colleagues discovered that the level of HLA-DR expression was closely linked to the degree of malignancy in gliomas, indicating that HLA-DR could be used as a biomarker for glioma malignancy (21). Our study has found a direct relationship between the expression of HLA-DR in tumors and ADC values. We also found that HLA-DR can be particularly intense in the stem cell component. It is noted that ADC values reflect tumor cellularity; precisely, low ADC values are related to higher tumor cellularity, which is associated with reduced glioma survival (41). By correlation analysis with the MRI parameter of ADC, we found that low ADC values corresponded to high tumor HLA-DR expression, thus corroborating previous findings that HLA-DR is effectively a biomarker of gliomas associated with malignancy. Our findings underline the translational relevance of our study that combines MRI with immunologic signature of GBM to gain more knowledge about this tumor.

Collectively, our findings make a compelling case for further research on the use of TAM subsets as diagnostic markers for GBMs. It should be noted that the interaction between cancer cells and immune cells requires further investigation. Therefore, coculture findings must be approached with caution.

Another limitation of our study is the lack of investigation of M1 functions in support of cancer stem cells and of evidence supporting the suggested ability of CD163/FKBP51s TAMs to migrate to brain areas far from the tumor. In addition, we have not considered advanced MRI methods that could provide more information about the TME. Several advanced radiographic techniques are emerging for studying immune cells within the GBM TME. Radiomics extracts and analyzes many quantitative features from medical images that can predict the presence and density of immune cells within the TME (42). PET has significantly advanced our understanding of neuroinflammation thanks to tracers that selectively bind to activated microglia and macrophages (43). Immuno-PET, which conjugates radionucleotides with molecules targeting immune cells, is in development for immune cell imaging and the evaluation of immunotherapies (43). Ferumoxytol, an intravenous iron oxide nanoparticle-based contrast agent used in MRI, visualizes immune infiltration within the brain tumor by TAM accumulation (44). However, these techniques are partly in a preclinical stage and do not fully resolve critical issues of morphologic MRI, also they need expert neuroradiologists.

The current study is groundbreaking in revealing that PB can be considered a surrogate liquid biopsy for patients with GBM. By identifying the similarity in the composition of TAMs between the TME and PB, this study has provided proof of reliable biomarkers that can be used for noninvasive clinical evaluation. Not only that, but this study has also identified FKBP51s as a prognostic biomarker of GBM tumors.

Although the study needs to be expanded, the results are original and innovative. They provide a promising breakthrough in the routine diagnostic protocols for patients with GBM. With the implementation of TAM as reliable biomarkers, clinicians could enhance their evaluation protocols and improve patient outcomes. This study is a stepping stone to transforming GBM diagnosis and monitoring.

## Supplementary Material

Supplementary Methods Details on plasmids, SiRNAs, antibodies, flow cytometry analysis, qPCR primers

Supplementary immunophenotyping This supplementary contains row data of immunophenotyping

Supplementary Figure S1 Flow chart of study population. Thirty-7 patients receiving diagnosis of glioblastoma were consecutively enrolled: 28 had primary tumors and 9 recurrences. Of 37 GB patients, 33 were included in the heatmap analysis, whereas 4 were excluded because of incomplete immunophenotyping of TME and/or peripheral blood, as also detailed in Table S1.

Supplementary Figure S2 Values of MFI of HLA-DR expression in TME-TAMs.

Supplementary Figure S3 Immunoblot (IB) of pSTAT3 expression levels upon FKBP51s modulation in glioblastoma cell lines. (a) IB of U87 cell transfected with Flag-FKBP51, Flag-FKBP51s or correspondent EV as control. The spliced FKBP51s (short), but not the canonical FKBP51 (long), increased pSTAT3 levels. (b) IB of D54 cell transfected with siFKBP51s or a non-silencing RNA (NS) as control. FKBP51s silencing reduced pSTAT 3 levels (c) IB of GB83 cells transfected with siFKBP51s or a non-silencing RNA (NS) as control and with Flag-FKBP51s or correspondent EV as control. Densitometric analysis of the two blots are shown. Bands were quantitated by densitometry, using ImageJ 1.42q for Macintosh. Integrated optical densities (ODs) were normalized to a relative housekeeping gene and expressed as fold increase, arbitrarily using the control of each experiment as reference sample (expression =1).

Supplementary Figure S4 IB of the expression levels of FKBP51s in 7 GB tumor cell lysates with the respective densitometric analysis of IB. Bands were quantitated by densitometry, using ImageJ 1.42q for Macintosh. Integrated ODs were normalized to two relative housekeeping genes (G3PDH and Tubulin).

Supplementary Figure S5 Cytokine levels in the sera from the two patients’ groups. Beyond the nominal p-values, the adjusted p-values by FDR method are also calculated and reported.

Supplementary Figure S6 Analysis by qPCR of the mRNA levels of the stemness markers CD133, EPHA2, NANOG, OCT 3 and 4, SNAIL, SOX2, ZEB1 in GB138 and GB83 spheroids (grey), compared to correspondent cells grown in adhesion (black).

Supplementary Figure S7 (a) Macrophage gating by CD45. (b) Representative flow cytometry histograms of CD163/FKBP51s expression on: M0 (no coculture), top; CD45-gated macrophages in cocultures with adherent GB138 (CC GB138), medium; or with spheroids GB138 (CC GB138 spheroids), bottom; the respective isotype antibodies are also shown on the left. (c) A representative flow cytometry histogram of HLA-DR expression in the same cells. (d) Analysis by qPCR of the mRNA levels of IL-10 and IL-4 in spheroids obtained by GB138 and GB83 cell lines, compared to each differentiated counterpart. (f) A representative gating of CD163/FKBP51s+(R1) and CD163/FKBP51s- (R2) macrophages. G) Flow cytometry histograms of pSTAT6 expression in gated R1 and R2 macrophages.

Supplementary Figure S8 Comparison of flow cytometry data of TME (upper) and PB (lower) between primary tumors (black columns) and recurrences (red columns). Only a difference (*) in HLA-DR and CD206 TME-TAMs (higher in primary tumors than recurrences) was registered. No difference in PB-TAMs counts was relieved.

Supplementary Figure S9 Callosal infiltration and Immunophenotype of TME and peripheral blood. Graphical representation of flow cytometry data of TME (graphs on the left) and peripheral blood (graphs on the right) from primary tumors (upper) and recurrences (lower). No CCI, black histograms; CCI, red histograms. Significant results (Mann Whitney) are underlined in red.

Supplementary Figure S10 ADC values and Immunophenotype of TME and peripheral blood. Graphical representation (linear regression) of flow cytometry data of TME (graphs on the left) and peripheral blood (graphs on the right) from primary tumors and recurrences. Significant result is underlined in red.

Supplementary Figure S11 Midline shift and Immunophenotype of TME and peripheral blood. Graphical representation of flow cytometry data of TME (graphs on the left) and peripheral blood (graphs on the right) from primary tumors (upper) and recurrences (lower). No midline shift, black histograms; midline shift, red histograms. P values are calculated using Mann Whitney test and unpaired T test with Welch correction.Significant results are underlined in red.

Supplementary Figure S12 Ependymal enhancement (EE) and Immunophenotype of TME and peripheral blood. Graphical representation of flow cytometry data of TME (graphs on the left) and peripheral blood (graphs on the right) from primary tumors (upper) and recurrences (lower). No EE, black histograms; EE, red histograms. P values are calculated using Mann Whitney test. The number of primary tumors with EE was <3.

Supplementary Figure S13 Tumor volume and Immunophenotype of TME and peripheral blood. Graphical representation (linear regression) of flow cytometry data of TME (graphs on the left) and peripheral blood (graphs on the right). Significant results are underlined in red.

Supplementary Figure S14 Necrosis score and Immunophenotype of TME and peripheral blood. Graphical representation of flow cytometry data of TME (graphs on the left) and peripheral blood (graphs on the right) from primary tumors. Patient were divided into 3 groups, NS=0,1 (black histogram), NS=2 (blue histogram), NS=3 (red histogram). Significant results (one way ANOVA) are underlined in red.

Supplementary Figure S15 ITSS score and Immunophenotype of TME and peripheral blood. Graphical representation of flow cytometry data of TME (graphs on the left) and peripheral blood (graphs on the right) from primary tumors. Patient were divided into 3 groups, ITSS score=0,1 (black histogram), ITSS score=2 (blue histogram), ITSS score=3 (red histogram) and p values were calculated using one way ANOVA.

Supplementary Figure S16 Vasogenic edema and Immunophenotype of TME and peripheral blood. Graphical representation of flow cytometry data of TME (graphs on the left) and peripheral blood (graphs on the right) from primary tumors. No Vasogenic edema, black histograms; Vasogenic edema, red histograms. P values are calculated using Mann Whitney test.

Table S1 Patients’ details

Table S2 Tumor FKBP51-expression does not affect the composition of TME in CD163 or HLA- DR-TAMs nor does it influence tumor HLA-DR expression. Pearson r coefficient and p values are indicated for each variable.

Table S3 Tumor FKBP51-expression does not affect the counts of the indicated peripheral blood TAM phenotypes nor does it influence CD4 and CD8 T lymphocytes’ counts. Pearson r coefficient and p values are indicated for each variable.

Table S4 Tumor FKBP51s expression and MRI features.

Table S5 Tumor volume and other MRI features: Pearson r coefficient and p values are indicated for each variable. Tumor volume is associated with midline shift.

Table S6 Necrosis score and immunophenotyping values: Pearson r coefficient and p values are indicated for each variable. Linear correlation of necrosis score with tumor PDL-1 expression and PDL-1/FKBP51s PB-TAMs.

Table S7 Necrosis score in relation to other MRI features: Pearson r coefficient and p values are indicated for each variable. Correlation between necrosis score and ITSS.

Table S8 ITSS score and immunophenotyping values: Pearson r coefficient and p values are indicated for each variable.
