# Supplementary material for: The FKBP51s Splice Isoform Predicts Unfavorable Prognosis in Patients with Glioblastoma
Source: Cancer Res Commun. 2024 May 16;4(5):1296–306. doi: 10.1158/2767-9764.CRC-24-0083 (PMC11097923; doi:10.1158/2767-9764.CRC-24-0083)
Supplement: Supplementary Methods — Details on plasmids, SiRNAs, antibodies, flow cytometry analysis, qPCR primers [file crc-24-0083-s01.pdf]

Plasmids and SiRNA

True-ORF-Myc-DDK (Flag)-tagged human FKBP51-transcript variant 1 (canonical FKBP51) and FKBP51-transcript variant 4 (FKBP51s) were purchased from OriGene Technologies (Rockville, Maryland, USA) (16). The relative empty vector (EV) was also transfected to generate control cells. No Sense RNA (NS RNA, Qiagen, Valencia, California, USA). For FKBP51s silencing, a mix of 3 siRNAs made of siRNA #1 and #2 targeting the 3′-coding region (between 700 and 1100 bp), and siRNA #3 targeting the 3′-UTR region (between 5200 and 5800 bp), was used as described previously (18, 19), which was custom produced by Qiagen.

List of antibodies for immunophenotyping

Cell surface antibodies: anti-CD14- Peridinin chlorophyll protein (PerCP) (TÜK4 clone; Miltenyi Biotec, Bergisch Gladbach, Germany), anti-PD-L1-phycoerythrin (PE) (MIH1 clone; eBioscience, Thermo Fisher Scientific, Waltham, Massachusetts, USA), anti-CD163- Allophycocyanin (APC) (GHI/61 clone; Molecular Probes, Thermo Fisher Scientific) anti-CD80-APC (MEM-233 clone; Immunotools, Friesoythe, Germany), Anti-Hu-CD36-Fluorescein-5-isothiocyanate (FITC) (NL07 clone; Invitrogen-Thermo Fisher Scientific), Anti-Hu HLA-DR-PE (L243 clone; Invitrogen, Thermo Fisher Scientific). anti- CD4-PerCP (VIT4 clone, Miltenyi Biotec), anti-CD3-PE (UCHT1 clone; BD Pharmingen), Anti-Hu CD169-PE (7-239 clone; Invitrogen, Thermo Fisher Scientific), Anti-Hu CD206-PE (19.2 Clone; Invitrogen-Thermo Fisher Scientific), Anti-Hu CD68-PE (Y1/82A clone; Immunotools), Anti-Hu CD45-APC (2D1 clone Invitrogen), Anti-Hu CD8-APC (UCHT-4 clone Immunotools).

Intracellular staining: anti-Hu/Mo Arginase1-AlexaFluor488 (A1exF5 clone; Invitrogen, Thermo Fisher Scientific), anti-Foxp3-PE (PCH101 clone, eBioscienceTM, Thermo Fisher Scientific) and anti-FKBP51s using anti-FKBP51s antibody conjugated with the 5-carboxyfluorescein (FAM) (16).

Indirect immunofluorescence anti-phospho-STAT6 (Tyr 641) (#93615, Cell Signaling Technology, Massachusetts, USA), Goat anti-Rabbit IgG (H+L) Cross-Adsorbed Secondary Antibody, FITC (F-2765, Invitrogen, Thermo Fisher Scientific).

Flow cytometry analysis of tumor samples

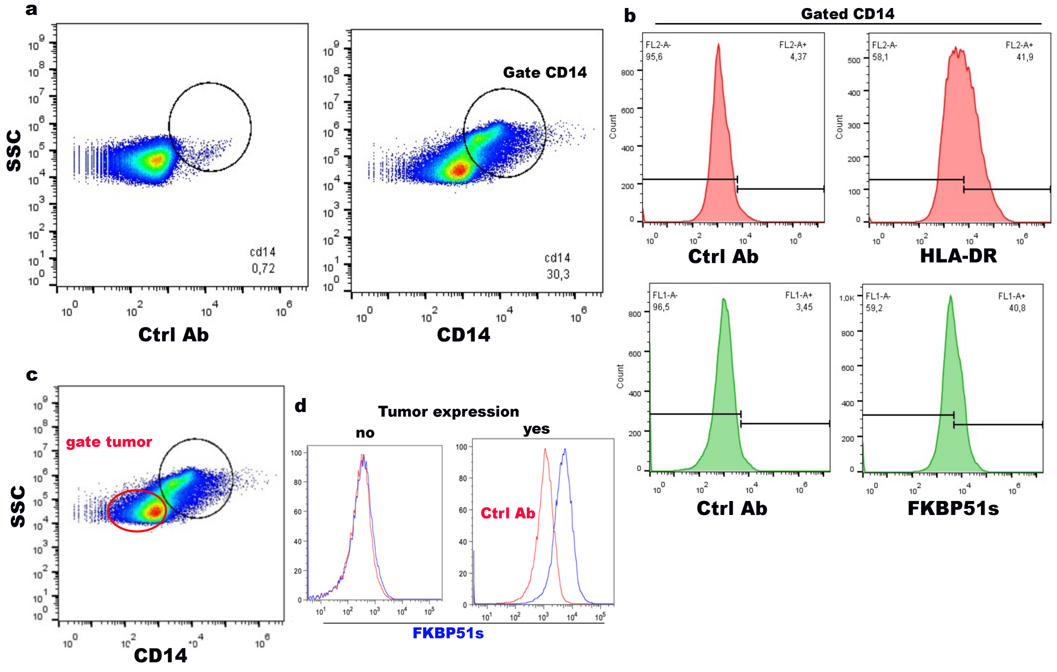

Representative histograms of flow cytometry analysis of TME. (a) Gating of tumor immune infiltrate according to CD14/SSc parameters. (b) Histograms of HLA-DR (top) and FKBP51s (bottom) expression in CD14 gated cells, with the respective isotype antibodies. The right bar indicates the proportion of positive cells (c) A representative gating of tumor cells by CD14

exclusion. (d) Representative histograms of FKBP51s negative (left) and positive (right) tumors shown in overlay with control antibody. Mean fluorescence intensity (MFI) of the specific marker served to quantify expression.

Multiplex cytokine kit

GB serum samples were centrifugated at 10,000×g for 10 minutes at 4°C and supernatants were harvested and processed as follows: 25 µL of the sample was diluted 1:8 with MACSPlex buffer (MPB) to a total volume of 200 µL and loaded onto wells of a pre-wet and drained MACSPlex 96-well 0.22 µm filter plate before 20 µL of MACSPlex Cytokine 12 Capture Beads. Flow cytometric analysis was performed with a MACSQuant Analyzer 10 flow cytometer (Miltenyi Biotec) by using the built-in 96-well plate reader. A standard curve was used to calculate the concentration of each cytokine on the MACSQuant Analyzer X. All samples were automatically mixed immediately before 70-100 µL were loaded to and acquired by the instrument, resulting in approximately 7,000-12,000 single bead events being recorded per well. Data were analyzed using the Express Mode option of the MACSQuantify 2.8 software (Miltenyi Biotec).

qPCR oligo sequences

hBACT-Fw: 5′-CGAGGCCCAGAGCAAGAGAG-3′;
hBACT-Rev: 5′-CGGTTGGCCTTAGGGTTCAG-3′
hCD133-Fw: 5’-TTCTTGACCGACTGAGACCC-3’;
hCD133-Rev: 5’-CCAAGCACAGAGGGTCATTG-3’;
hEPHA2-Fw: 5’-ACTTCTTGTAGTAGACACGGACG-3’;
hEPHA2-Rev: 5’-AATAGAGAGGTTTGGCTTCCAGAC-3’;
hIL10-Fw: 5′-GGCACCCAGTCTGAGAACAG--3′;
hIL10-Rev: 5′-TGGCAACCCAGGTAACCCTTA-3′;
hIL4-Fw: TTGCTGCCTCCAAGAACACA;
hIL4-Rev: TCCAACGTACTCTGGTTGGC;
hIL17-Fw: 5’-CCAAAAGCCTGAGAGTTGCC-3’;
hIL17-Rev: 5’-TTGATGCAGCCCAAGTTCCT-3’;
hNANOG-Fw: 5′-CAAAGGCAAACAACCCACTT-3′;
hNANOG-Rev: 5′-TCTGGAACCAGGTCTTCACC-3′;
hOCT 3/4-Fw: 5’-CGAAAGAGAAAGCGAACCAG-3’;
hOCT 3/4-Rev:5’-GCCGGTTACAGAACCACACT-3’;
h-SDHA-Fw: 5′-TGGGAACAAGAGGGCATCTG-3’;
h-SDHA-Rev: 5′-CCACCACTGCATCAAATTCATG-3′;
hSNAIL-Fw: 5’-AGTGGTTCTTCTGCGCTACT-3’;
hSNAIL-Rev: 5’-GGGCTGCTGGAAGGTAAACT-3’;
hSOX2-Fw: 5’-GCACATGAACGGCTGGAGCAACG-3’;
hSOX2-Rev: 5’-TGCTGCGAGTAGGACATGCTGTAGGG-3’;
hZEB1-Fw: 5’-CCAGACAGTGTTACCAGGGAGGAG-3’;
hZEB1-Rev: 5’-TGCCCTTCCTTTCCTGTGTCATCC-3’;
h-RPS18-Fw: 5′- CGATGCGGCGGCGTTATTC-3′;
h-RPS18-Rev-5′- TCTGTCAATCCTGTCCGTGTCC-3′
Validated IFNA qPCR primers were purchased from Qiagen (QT00201964: QuantiTect Primer Assay).

Antibodies for Immunoblot

Anti-phospho-STAT3 (Tyr 705) (rabbit polyclonal, GTX133464, GeneTex, Irvine, California, USA) (1:2000), anti-FKBP51 (rabbit polyclonal, NB100-68240, Novus Biological, Abingdon, UK); anti-FKBP51s (Romano S et al 2015), anti-phospho-STAT6 (Tyr 641) (rabbit polyclonal, #93615, Cell Signaling Technology, Inc. Massachusetts, USA), anti-Stat3 (mouse monoclonal, sc-8019, Santa

Cruz Biotechnology, California, USA), anti-Stat6 (mouse monoclonal, sc1689, Santa Cruz Biotechnology), anti-β-Actin (mouse monoclonal, 15G5A11/E2, Invitrogen, Thermo Fisher Scientific), anti-Vinculin (mouse monoclonal, sc-55465 Santa Cruz Biotechnology), anti-G3PDH (mouse monoclonal, sc-32233, Santa Cruz Biotechnology), anti-γ-tubulin (mouse monoclonal, T9026, Sigma-Aldrich).

Quantification of bands

Integrated optical densities of each analyzed protein were normalized to a relative housekeeping gene. Values were expressed as normalized ODs or as fold change of protein levels in the different samples in comparison with a control sample (expression =1).
