# Supplementary material for: The FKBP51s Splice Isoform Predicts Unfavorable Prognosis in Patients with Glioblastoma
Source: Cancer Res Commun. 2024 May 16;4(5):1296–306. doi: 10.1158/2767-9764.CRC-24-0083 (PMC11097923; doi:10.1158/2767-9764.CRC-24-0083)
Supplement: Supplementary Figure S1 — Flow chart of study population. Thirty-7 patients receiving diagnosis of glioblastoma were consecutively enrolled: 28 had primary tumors and 9 recurrences. Of 37 GB patients, 33 were included in the heatmap analysis, whereas 4 were excluded because of incomplete immunophenotyping of TME and/or peripheral blood, as also detailed in Table S1. [file crc-24-0083-s03.pdf]

Supplementary Figure S1

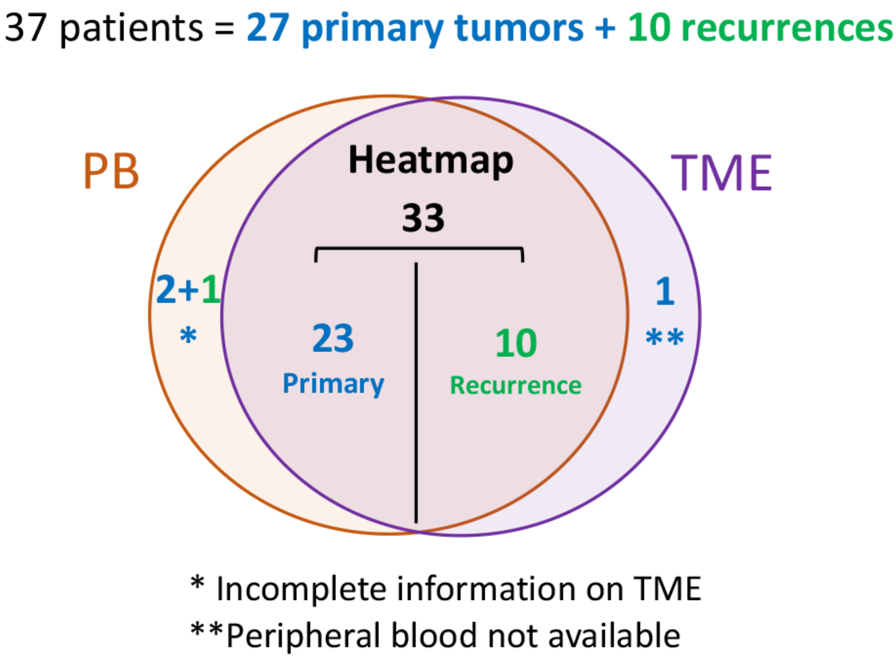

Fig S1. Flow chart of study population. Thirty-7 patients receiving diagnosis of glioblastoma were consecutively enrolled: 28 had primary tumors and 9 recurrences. Of 37 GB patients, 33 were included in the heatmap analysis, whereas 4 were excluded because of incomplete immunophenotyping of TME and/or peripheral blood, as also detailed in Table S1.
