# Supplementary figures and images for: The FKBP51s Splice Isoform Predicts Unfavorable Prognosis in Patients with Glioblastoma
Source: Cancer Res Commun. 2024 May 16;4(5):1296–306. doi: 10.1158/2767-9764.CRC-24-0083 (PMC11097923; doi:10.1158/2767-9764.CRC-24-0083)

Supplementary Figure S2

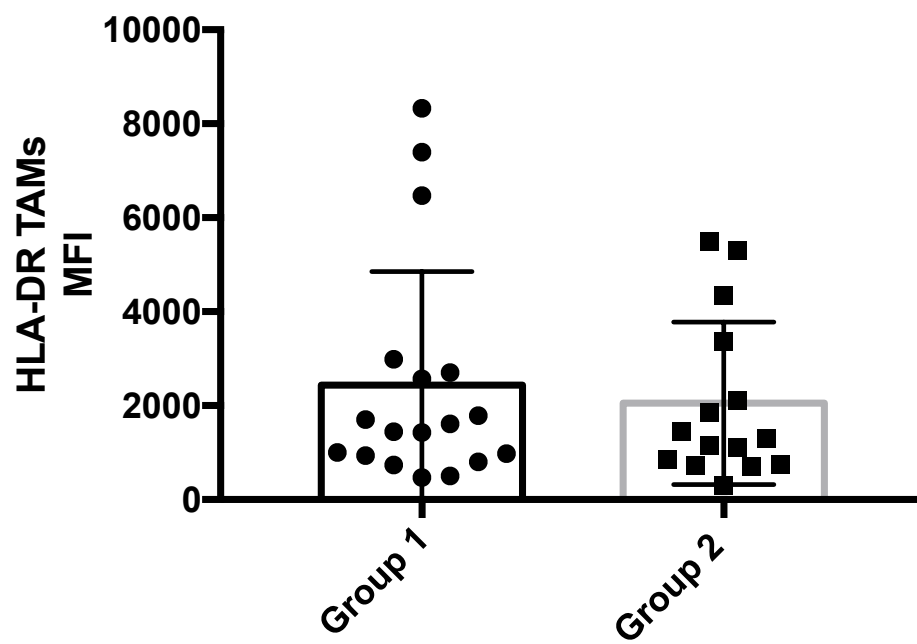

**Fig S2.** Values of MFI of HLA-DR expression in TME-TAMs.

Supplement: Supplementary Figure S2 — Values of MFI of HLA-DR expression in TME-TAMs. [file crc-24-0083-s04.pdf]
