# Supplementary material for: The FKBP51s Splice Isoform Predicts Unfavorable Prognosis in Patients with Glioblastoma
Source: Cancer Res Commun. 2024 May 16;4(5):1296–306. doi: 10.1158/2767-9764.CRC-24-0083 (PMC11097923; doi:10.1158/2767-9764.CRC-24-0083)
Supplement: Supplementary Figure S3 — Immunoblot (IB) of pSTAT3 expression levels upon FKBP51s modulation in glioblastoma cell lines. (a) IB of U87 cell transfected with Flag-FKBP51, Flag-FKBP51s or correspondent EV as control. The spliced FKBP51s (short), but not the canonical FKBP51 (long), increased pSTAT3 levels. (b) IB of D54 cell transfected with siFKBP51s or a non-silencing RNA (NS) as control. FKBP51s silencing reduced pSTAT 3 levels (c) IB of GB83 cells transfected with siFKBP51s or a non-silencing RNA (NS) as control and with Flag-FKBP51s or correspondent EV as control. Densitometric analysis of the two blots are shown. Bands were quantitated by densitometry, using ImageJ 1.42q for Macintosh. Integrated optical densities (ODs) were normalized to a relative housekeeping gene and expressed as fold increase, arbitrarily using the control of each experiment as reference sample (expression =1). [file crc-24-0083-s05.pdf]

Supplementary Figure S3

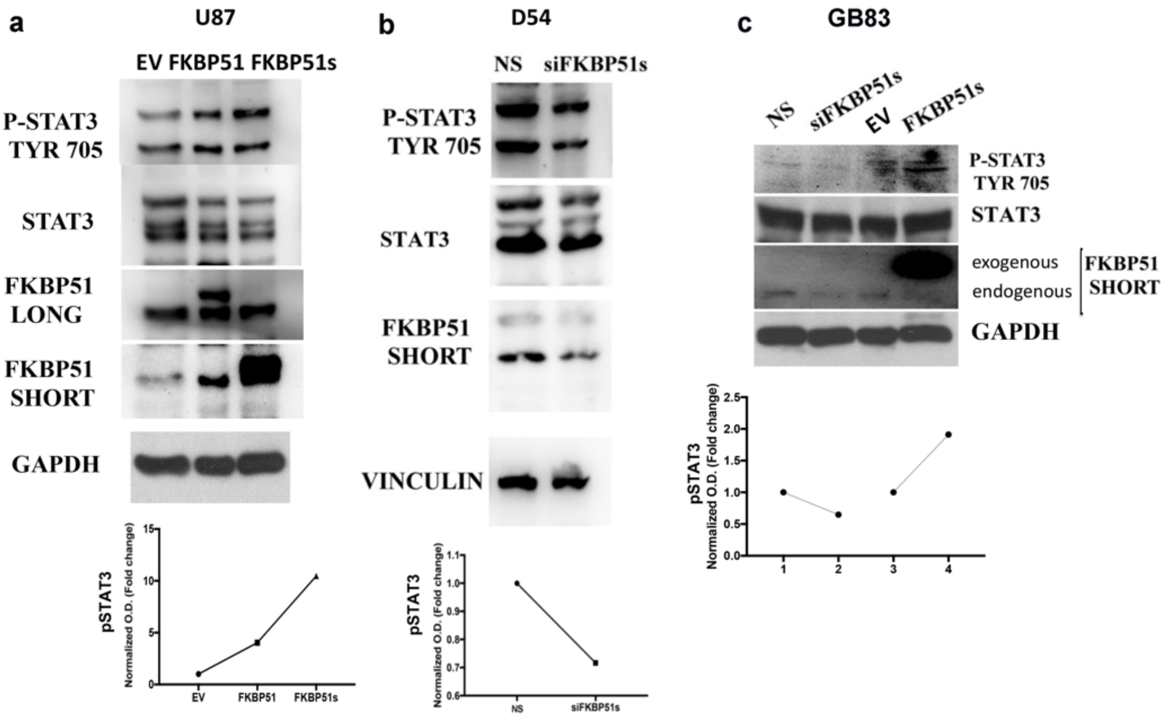

Fig S3. Immunoblot (IB) of pSTAT3 expression levels upon FKBP51s modulation in glioblastoma cell lines. (a) IB of U87 cell transfected with Flag-FKBP51, Flag-FKBP51s or correspondent EV as control. The spliced FKBP51s (short), but not the canonical FKBP51 (long), increased pSTAT3 levels. (b) IB of D54 cell transfected with siFKBP51s or a non-silencing RNA (NS) as control. FKBP51s silencing reduced pSTAT 3 levels (c) IB of GB83 cells transfected with siFKBP51s or a non-silencing RNA (NS) as control and with Flag-FKBP51s or correspondent EV as control. Densitometric analysis of the two blots are shown. Bands were quantitated by densitometry, using ImageJ 1.42q for Macintosh. Integrated optical densities (ODs) were normalized to a relative housekeeping gene and expressed as fold increase, arbitrarily using the control of each experiment as reference sample (expression =1).
