# Supplementary material for: The FKBP51s Splice Isoform Predicts Unfavorable Prognosis in Patients with Glioblastoma
Source: Cancer Res Commun. 2024 May 16;4(5):1296–306. doi: 10.1158/2767-9764.CRC-24-0083 (PMC11097923; doi:10.1158/2767-9764.CRC-24-0083)
Supplement: Supplementary Figure S4 — IB of the expression levels of FKBP51s in 7 GB tumor cell lysates with the respective densitometric analysis of IB. Bands were quantitated by densitometry, using ImageJ 1.42q for Macintosh. Integrated ODs were normalized to two relative housekeeping genes (G3PDH and Tubulin). [file crc-24-0083-s06.pdf]

Supplementary Figure S4

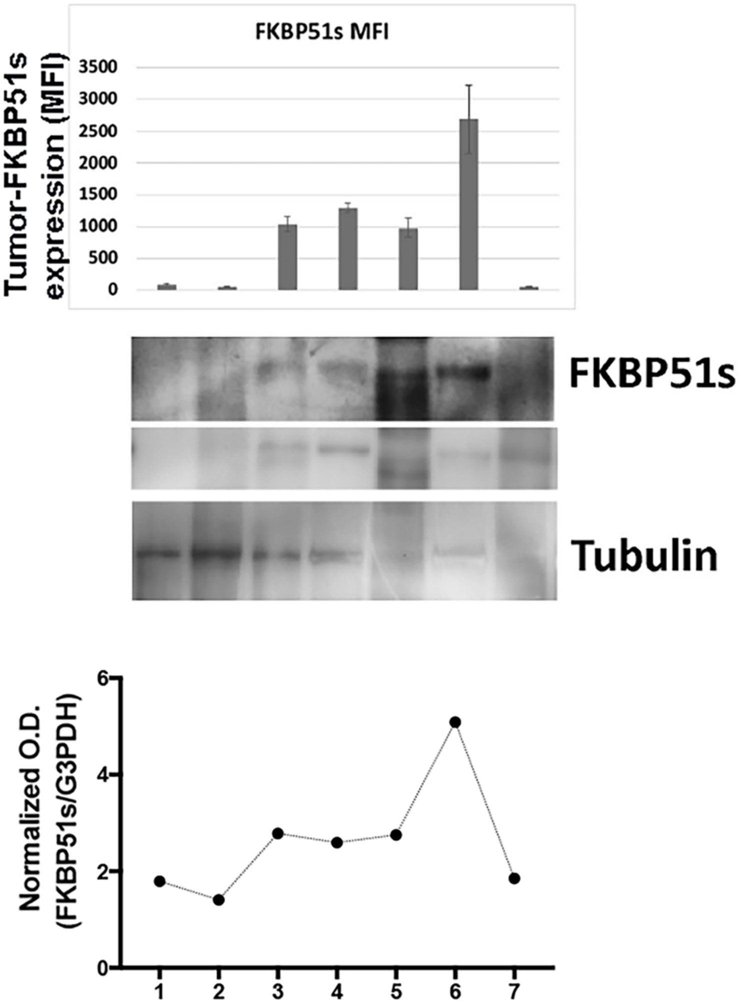

Fig S4. IB of the expression levels of FKBP51s in 7 GB tumor cell lysates with the respective densitometric analysis of IB. Bands were quantitated by densitometry, using ImageJ 1.42q for Macintosh. Integrated ODs were normalized to two relative housekeeping genes (G3PDH and Tubulin).
