# Supplementary material for: The FKBP51s Splice Isoform Predicts Unfavorable Prognosis in Patients with Glioblastoma
Source: Cancer Res Commun. 2024 May 16;4(5):1296–306. doi: 10.1158/2767-9764.CRC-24-0083 (PMC11097923; doi:10.1158/2767-9764.CRC-24-0083)
Supplement: Supplementary Figure S5 — Cytokine levels in the sera from the two patients’ groups. Beyond the nominal p-values, the adjusted p-values by FDR method are also calculated and reported. [file crc-24-0083-s07.pdf]

Supplementary Figure S5

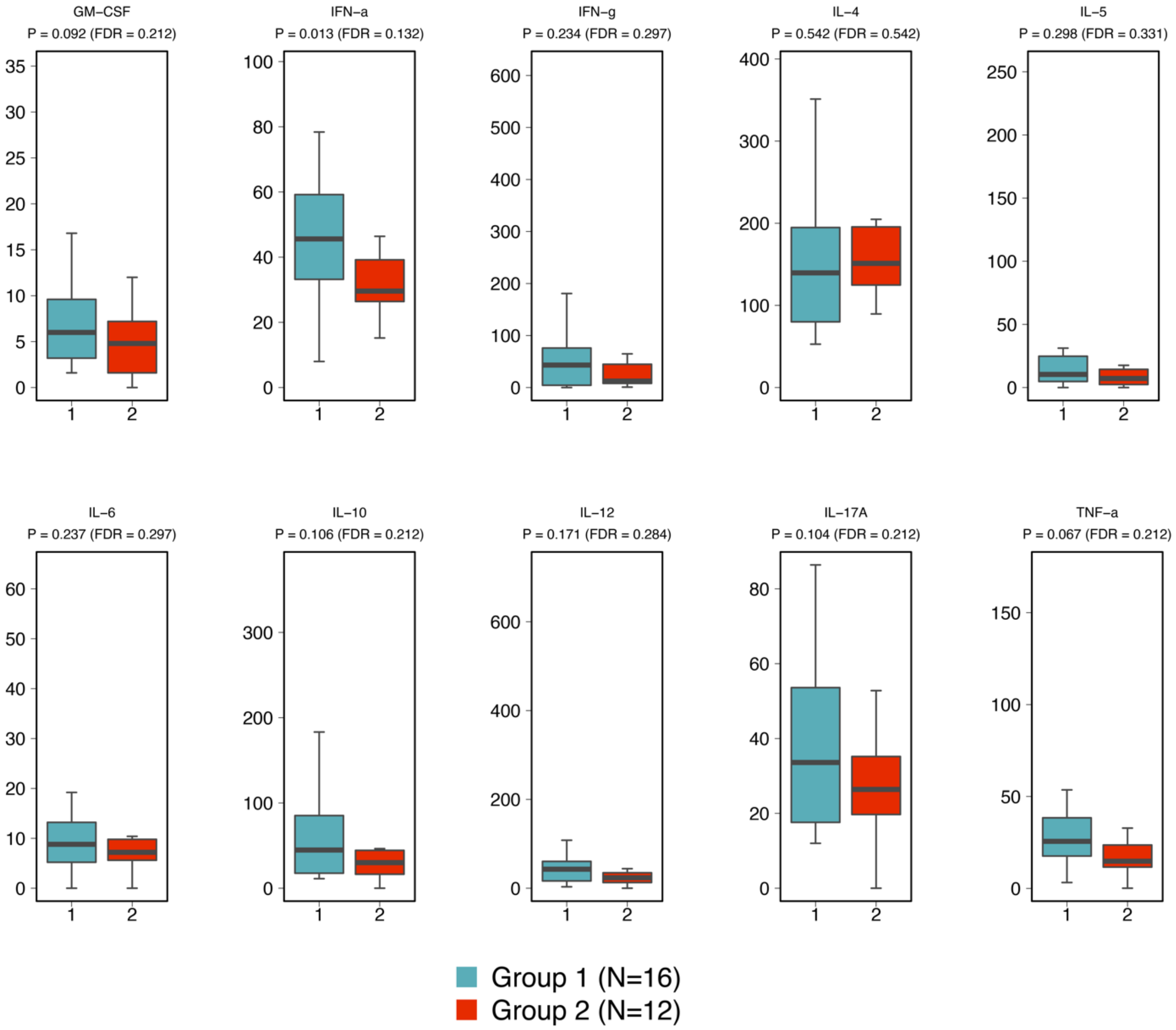

Fig S5 Cytokine levels in the sera from the two patients' groups. Beyond the nominal p-values, the adjusted p-values by FDR method are also calculated and reported.
