# Supplementary material for: The FKBP51s Splice Isoform Predicts Unfavorable Prognosis in Patients with Glioblastoma
Source: Cancer Res Commun. 2024 May 16;4(5):1296–306. doi: 10.1158/2767-9764.CRC-24-0083 (PMC11097923; doi:10.1158/2767-9764.CRC-24-0083)
Supplement: Supplementary Figure S6 — Analysis by qPCR of the mRNA levels of the stemness markers CD133, EPHA2, NANOG, OCT 3 and 4, SNAIL, SOX2, ZEB1 in GB138 and GB83 spheroids (grey), compared to correspondent cells grown in adhesion (black). [file crc-24-0083-s08.pdf]

Supplementary Figure S6

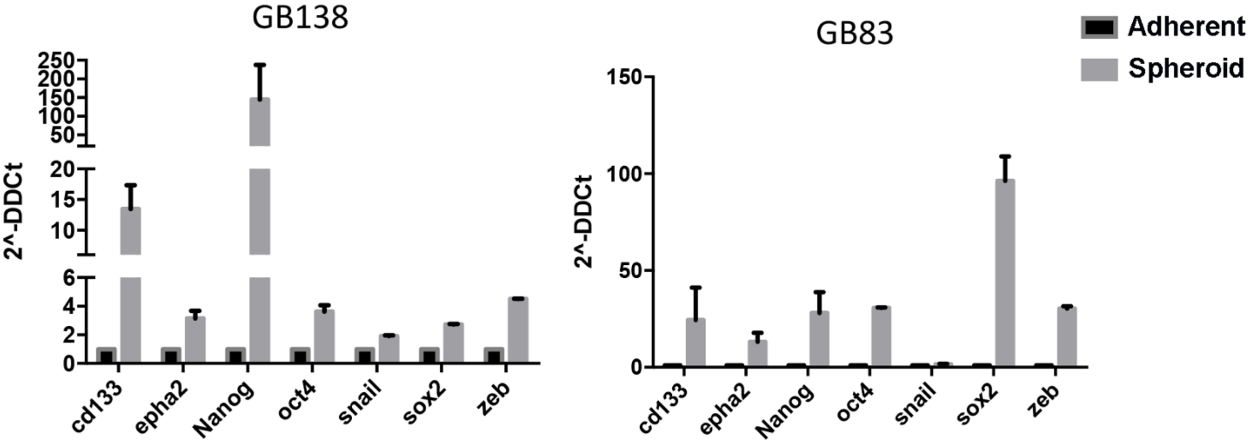

Fig S6. Analysis by qPCR of the mRNA levels of the stemness markers CD133, EPHA2, NANOG, OCT 3 and 4, SNAIL, SOX2, ZEB1 in GB138 and GB83 spheroids (grey), compared to correspondent cells grown in adhesion (black).
