# Supplementary material for: The FKBP51s Splice Isoform Predicts Unfavorable Prognosis in Patients with Glioblastoma
Source: Cancer Res Commun. 2024 May 16;4(5):1296–306. doi: 10.1158/2767-9764.CRC-24-0083 (PMC11097923; doi:10.1158/2767-9764.CRC-24-0083)
Supplement: Supplementary Figure S7 — (a) Macrophage gating by CD45. (b) Representative flow cytometry histograms of CD163/FKBP51s expression on: M0 (no coculture), top; CD45-gated macrophages in cocultures with adherent GB138 (CC GB138), medium; or with spheroids GB138 (CC GB138 spheroids), bottom; the respective isotype antibodies are also shown on the left. (c) A representative flow cytometry histogram of HLA-DR expression in the same cells. (d) Analysis by qPCR of the mRNA levels of IL-10 and IL-4 in spheroids obtained by GB138 and GB83 cell lines, compared to each differentiated counterpart. (f) A representative gating of CD163/FKBP51s+(R1) and CD163/FKBP51s- (R2) macrophages. G) Flow cytometry histograms of pSTAT6 expression in gated R1 and R2 macrophages. [file crc-24-0083-s09.pdf]

Supplementary Figure S7

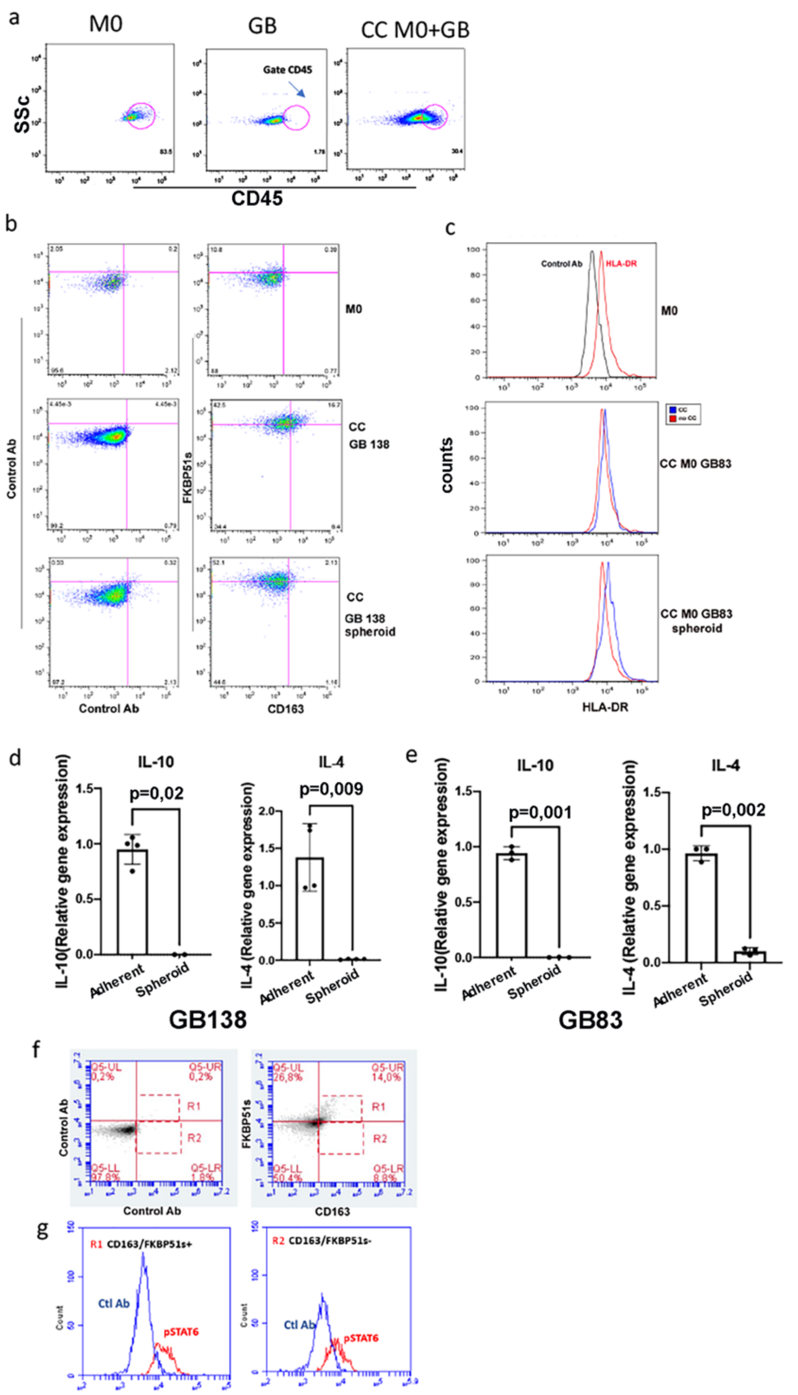

Fig S7. (a) Macrophage gating by CD45. (b) Representative flow cytometry histograms of CD163/FKBP51s expression on: M0 (no coculture), top; CD45-gated macrophages in cocultures with adherent GB138 (CC GB138), medium; or with spheroids GB138 (CC GB138 spheroids), bottom; the respective isotype antibodies are also shown on the left. (c) A representative flow cytometry histogram of HLA-DR expression in the same cells. (d) Analysis by qPCR of the mRNA levels of IL-10 and IL-4 in spheroids obtained by GB138 and GB83 cell lines, compared to each differentiated counterpart. (f) A representative gating of CD163/FKBP51s^{+}(R1) and CD163/FKBP51s^{-} (R2) macrophages. G) Flow cytometry histograms of pSTAT6 expression in gated R1 and R2 macrophages.
