# Supplementary material for: The FKBP51s Splice Isoform Predicts Unfavorable Prognosis in Patients with Glioblastoma
Source: Cancer Res Commun. 2024 May 16;4(5):1296–306. doi: 10.1158/2767-9764.CRC-24-0083 (PMC11097923; doi:10.1158/2767-9764.CRC-24-0083)
Supplement: Supplementary Figure S8 — Comparison of flow cytometry data of TME (upper) and PB (lower) between primary tumors (black columns) and recurrences (red columns). Only a difference (*) in HLA-DR and CD206 TME-TAMs (higher in primary tumors than recurrences) was registered. No difference in PB-TAMs counts was relieved. [file crc-24-0083-s10.pdf]

Supplementary Figure S8

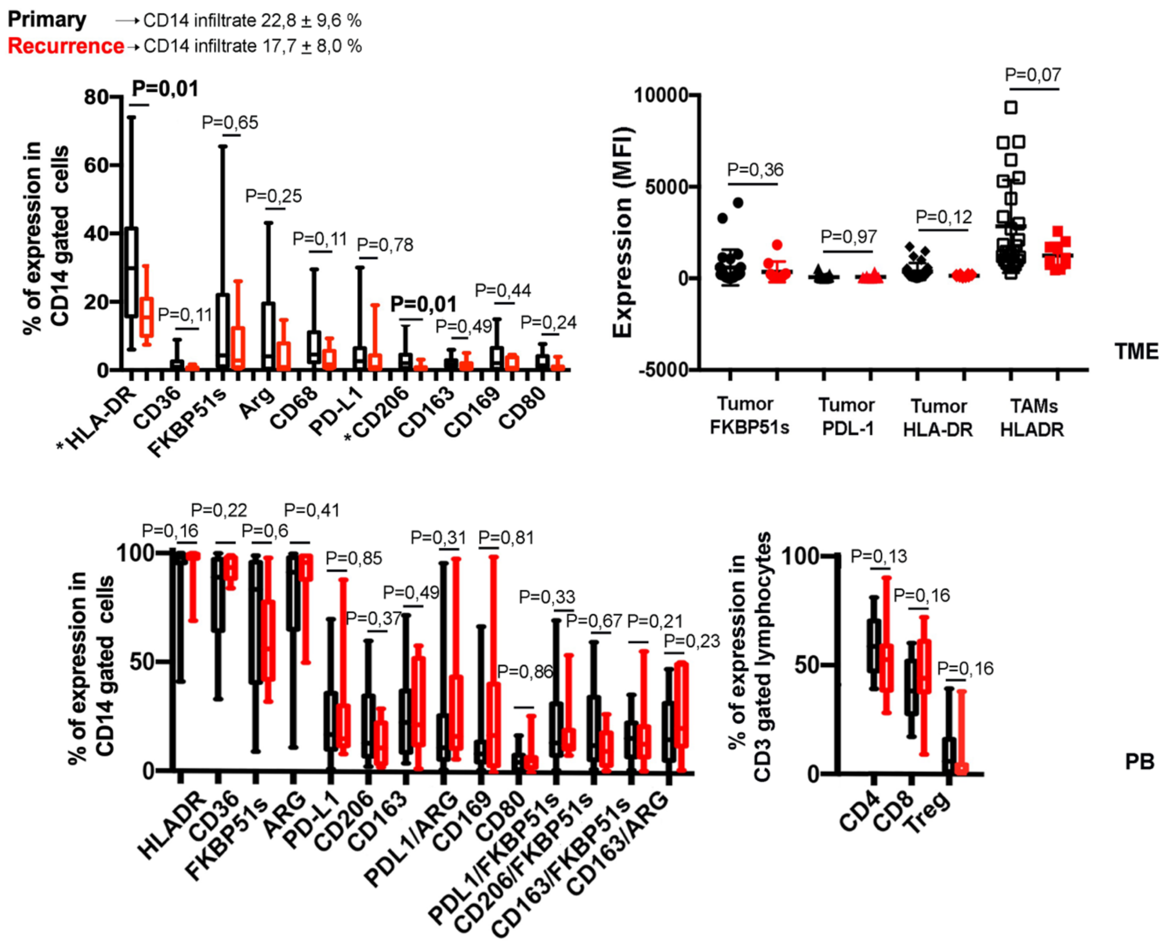

Fig S8 Comparison of flow cytometry data of TME (upper) and PB (lower) between primary tumors (black columns) and recurrences (red columns). Only a difference (*) in HLA-DR and CD206 TME-TAMs (higher in primary tumors than recurrences) was registered. No difference in PB-TAMs counts was relieved.
