# Supplementary material for: The FKBP51s Splice Isoform Predicts Unfavorable Prognosis in Patients with Glioblastoma
Source: Cancer Res Commun. 2024 May 16;4(5):1296–306. doi: 10.1158/2767-9764.CRC-24-0083 (PMC11097923; doi:10.1158/2767-9764.CRC-24-0083)
Supplement: Supplementary Figure S9 — Callosal infiltration and Immunophenotype of TME and peripheral blood. Graphical representation of flow cytometry data of TME (graphs on the left) and peripheral blood (graphs on the right) from primary tumors (upper) and recurrences (lower). No CCI, black histograms; CCI, red histograms. Significant results (Mann Whitney) are underlined in red. [file crc-24-0083-s11.pdf]

Supplementary Figure S9

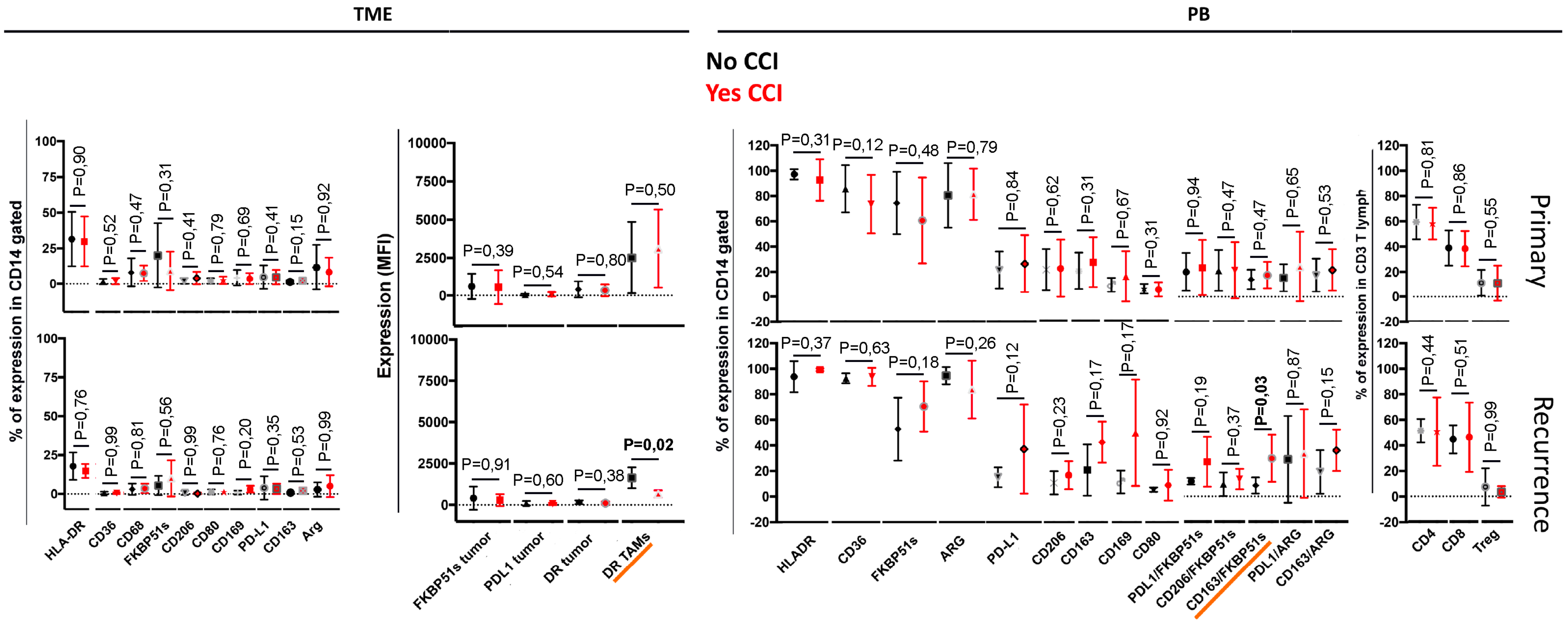

Fig S9. Callosal infiltration and Immunophenotype of TME and peripheral blood. Graphical representation of flow cytometry data of TME (graphs on the left) and peripheral blood (graphs on the right) from primary tumors (upper) and recurrences (lower). No CCI, black histograms; CCI, red histograms. Significant results (Mann Whitney) are underlined in red.
