# Supplementary material for: The FKBP51s Splice Isoform Predicts Unfavorable Prognosis in Patients with Glioblastoma
Source: Cancer Res Commun. 2024 May 16;4(5):1296–306. doi: 10.1158/2767-9764.CRC-24-0083 (PMC11097923; doi:10.1158/2767-9764.CRC-24-0083)
Supplement: Supplementary Figure S11 — Midline shift and Immunophenotype of TME and peripheral blood. Graphical representation of flow cytometry data of TME (graphs on the left) and peripheral blood (graphs on the right) from primary tumors (upper) and recurrences (lower). No midline shift, black histograms; midline shift, red histograms. P values are calculated using Mann Whitney test and unpaired T test with Welch correction.Significant results are underlined in red. [file crc-24-0083-s13.pdf]

Supplementary Figure S11

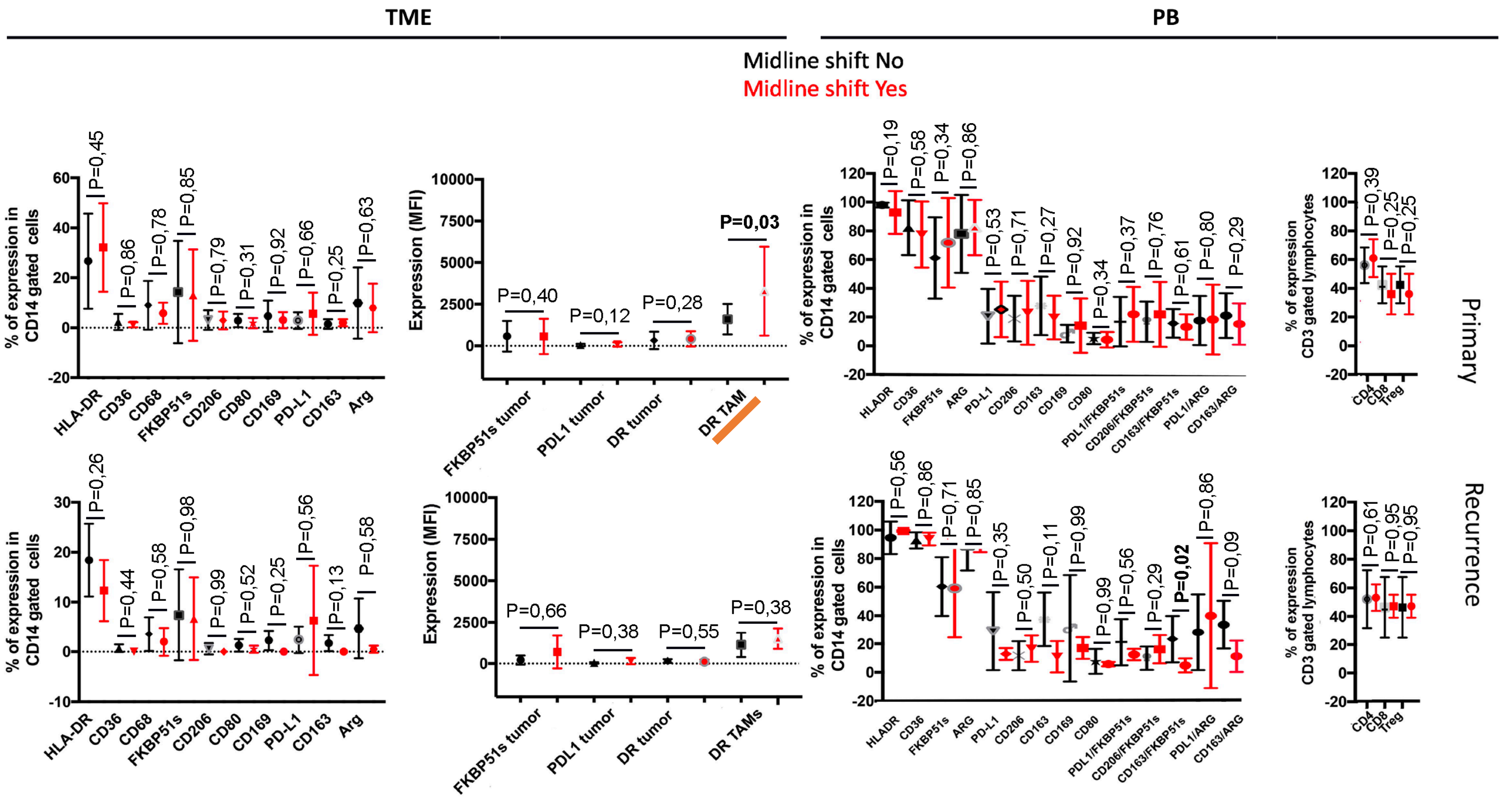

Fig S11. Midline shift and Immunophenotype of TME and peripheral blood. Graphical representation of flow cytometry data of TME (graphs on the left) and peripheral blood (graphs on the right) from primary tumors (upper) and recurrences (lower). No midline shift, black histograms; midline shift, red histograms. P values are calculated using Mann Whitney test and unpaired T test with Welch correction.Significant results are underlined in red.
