# Supplementary material for: The FKBP51s Splice Isoform Predicts Unfavorable Prognosis in Patients with Glioblastoma
Source: Cancer Res Commun. 2024 May 16;4(5):1296–306. doi: 10.1158/2767-9764.CRC-24-0083 (PMC11097923; doi:10.1158/2767-9764.CRC-24-0083)
Supplement: Supplementary Figure S12 — Ependymal enhancement (EE) and Immunophenotype of TME and peripheral blood. Graphical representation of flow cytometry data of TME (graphs on the left) and peripheral blood (graphs on the right) from primary tumors (upper) and recurrences (lower). No EE, black histograms; EE, red histograms. P values are calculated using Mann Whitney test. The number of primary tumors with EE was <3. [file crc-24-0083-s14.pdf]

Supplementary Figure S12

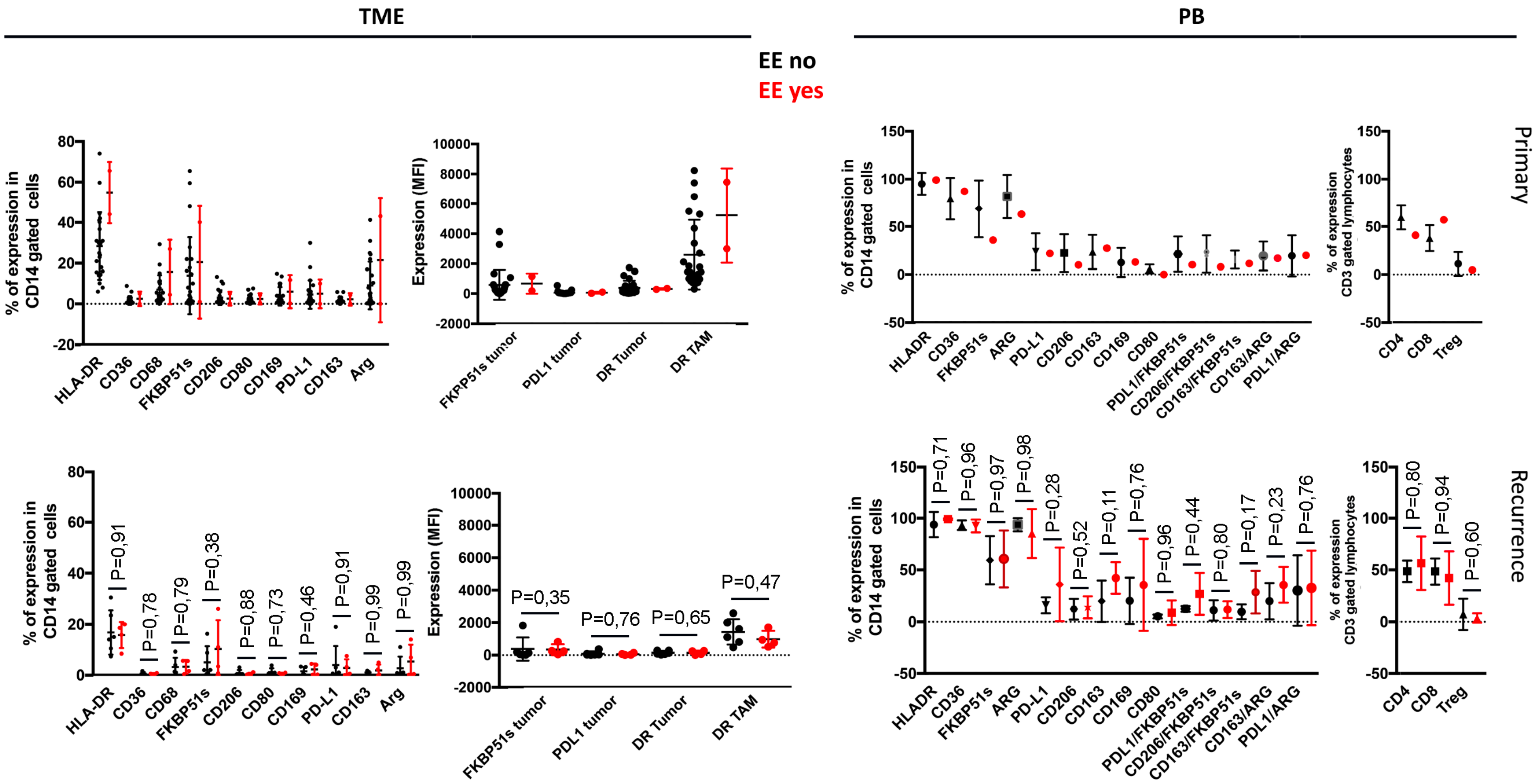

Fig S12. Ependymal enhancement (EE) and Immunophenotype of TME and peripheral blood. Graphical representation of flow cytometry data of TME (graphs on the left) and peripheral blood (graphs on the right) from primary tumors (upper) and recurrences (lower). No EE, black histograms; EE, red histograms. P values are calculated using Mann Whitney test. The number of primary tumors with EE was <3.
