# Supplementary material for: The FKBP51s Splice Isoform Predicts Unfavorable Prognosis in Patients with Glioblastoma
Source: Cancer Res Commun. 2024 May 16;4(5):1296–306. doi: 10.1158/2767-9764.CRC-24-0083 (PMC11097923; doi:10.1158/2767-9764.CRC-24-0083)
Supplement: Supplementary Figure S13 — Tumor volume and Immunophenotype of TME and peripheral blood. Graphical representation (linear regression) of flow cytometry data of TME (graphs on the left) and peripheral blood (graphs on the right). Significant results are underlined in red. [file crc-24-0083-s15.pdf]

Supplementary Figure S13

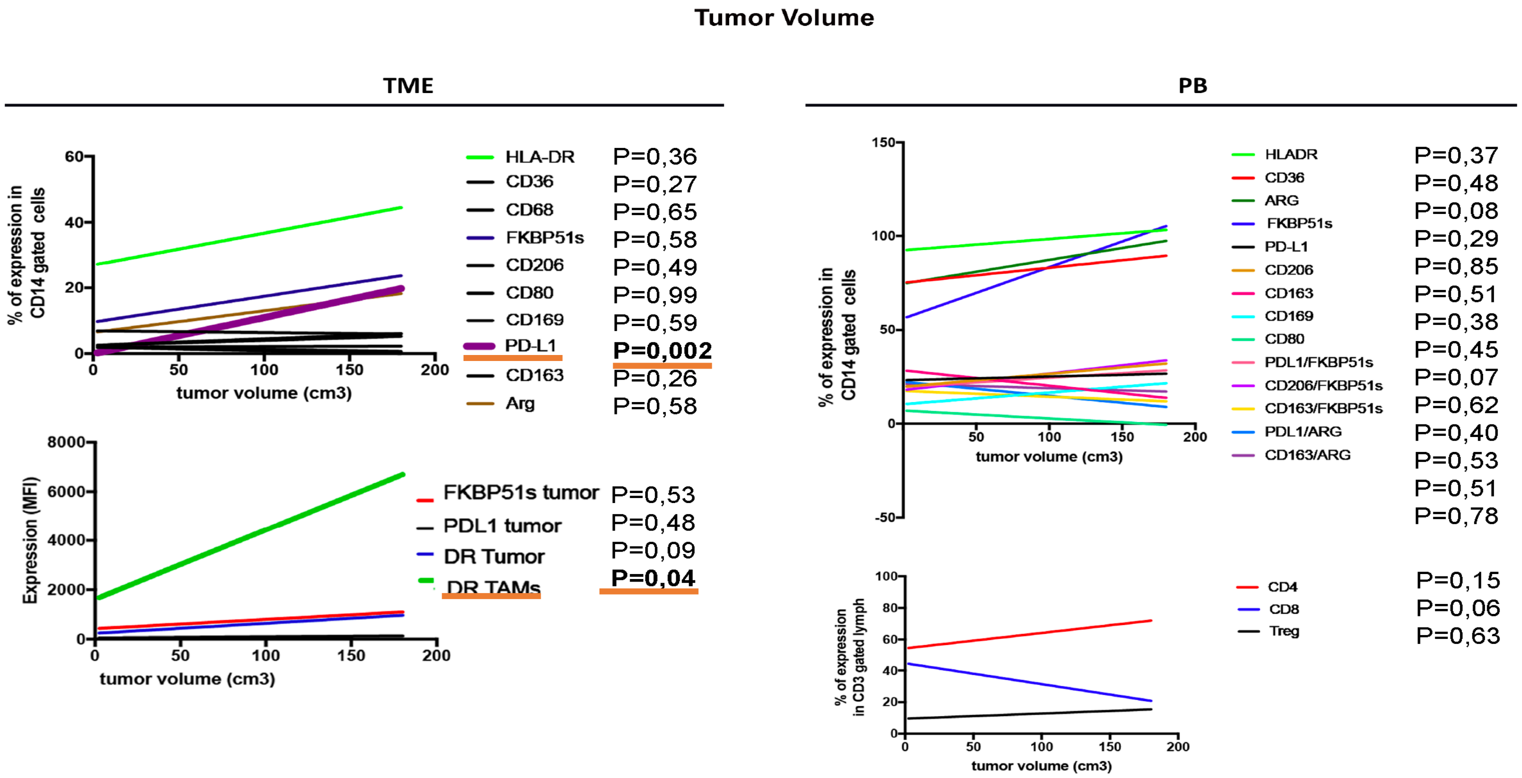

Fig S13. Tumor volume and Immunophenotype of TME and peripheral blood. Graphical representation (linear regression) of flow cytometry data of TME (graphs on the left) and peripheral blood (graphs on the right). Significant results are underlined in red.
