# Supplementary material for: The FKBP51s Splice Isoform Predicts Unfavorable Prognosis in Patients with Glioblastoma
Source: Cancer Res Commun. 2024 May 16;4(5):1296–306. doi: 10.1158/2767-9764.CRC-24-0083 (PMC11097923; doi:10.1158/2767-9764.CRC-24-0083)
Supplement: Supplementary Figure S14 — Necrosis score and Immunophenotype of TME and peripheral blood. Graphical representation of flow cytometry data of TME (graphs on the left) and peripheral blood (graphs on the right) from primary tumors. Patient were divided into 3 groups, NS=0,1 (black histogram), NS=2 (blue histogram), NS=3 (red histogram). Significant results (one way ANOVA) are underlined in red. [file crc-24-0083-s16.pdf]

Supplementary Figure S14

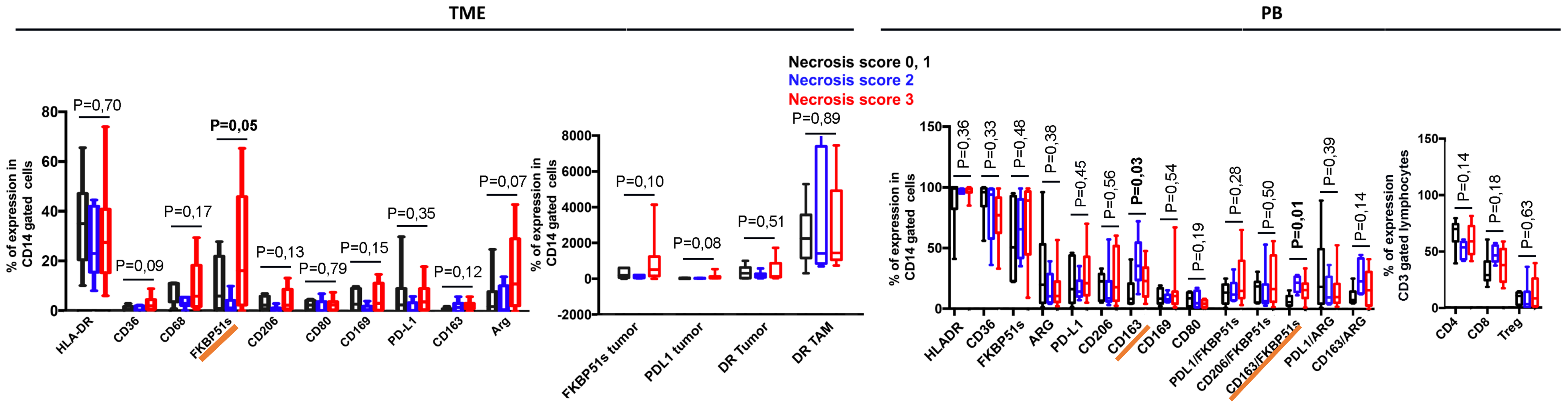

Fig S14. Necrosis score and Immunophenotype of TME and peripheral blood. Graphical representation of flow cytometry data of TME (graphs on the left) and peripheral blood (graphs on the right) from primary tumors. Patient were divided into 3 groups, NS=0,1 (black histogram), NS=2 (blue histogram), NS=3 (red histogram). Significant results (one way ANOVA) are underlined in red.
