# Supplementary material for: The FKBP51s Splice Isoform Predicts Unfavorable Prognosis in Patients with Glioblastoma
Source: Cancer Res Commun. 2024 May 16;4(5):1296–306. doi: 10.1158/2767-9764.CRC-24-0083 (PMC11097923; doi:10.1158/2767-9764.CRC-24-0083)
Supplement: Supplementary Figure S15 — ITSS score and Immunophenotype of TME and peripheral blood. Graphical representation of flow cytometry data of TME (graphs on the left) and peripheral blood (graphs on the right) from primary tumors. Patient were divided into 3 groups, ITSS score=0,1 (black histogram), ITSS score=2 (blue histogram), ITSS score=3 (red histogram) and p values were calculated using one way ANOVA. [file crc-24-0083-s17.pdf]

Supplementary Figure S15

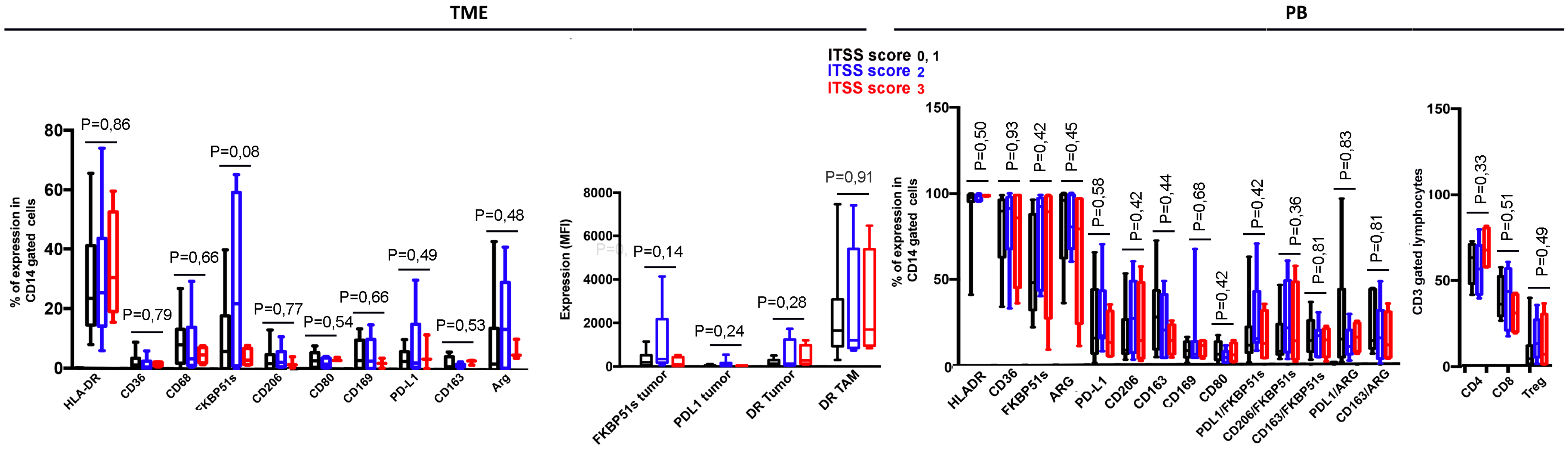

Fig S15. ITSS score and Immunophenotype of TME and peripheral blood. Graphical representation of flow cytometry data of TME (graphs on the left) and peripheral blood (graphs on the right) from primary tumors. Patient were divided into 3 groups, ITSS score=0,1 (black histogram), ITSS score=2 (blue histogram), ITSS score=3 (red histogram) and p values were calculated using one way ANOVA.
