# Supplementary material for: The FKBP51s Splice Isoform Predicts Unfavorable Prognosis in Patients with Glioblastoma
Source: Cancer Res Commun. 2024 May 16;4(5):1296–306. doi: 10.1158/2767-9764.CRC-24-0083 (PMC11097923; doi:10.1158/2767-9764.CRC-24-0083)
Supplement: Supplementary Figure S16 — Vasogenic edema and Immunophenotype of TME and peripheral blood. Graphical representation of flow cytometry data of TME (graphs on the left) and peripheral blood (graphs on the right) from primary tumors. No Vasogenic edema, black histograms; Vasogenic edema, red histograms. P values are calculated using Mann Whitney test. [file crc-24-0083-s18.pdf]

Supplementary Figure S16

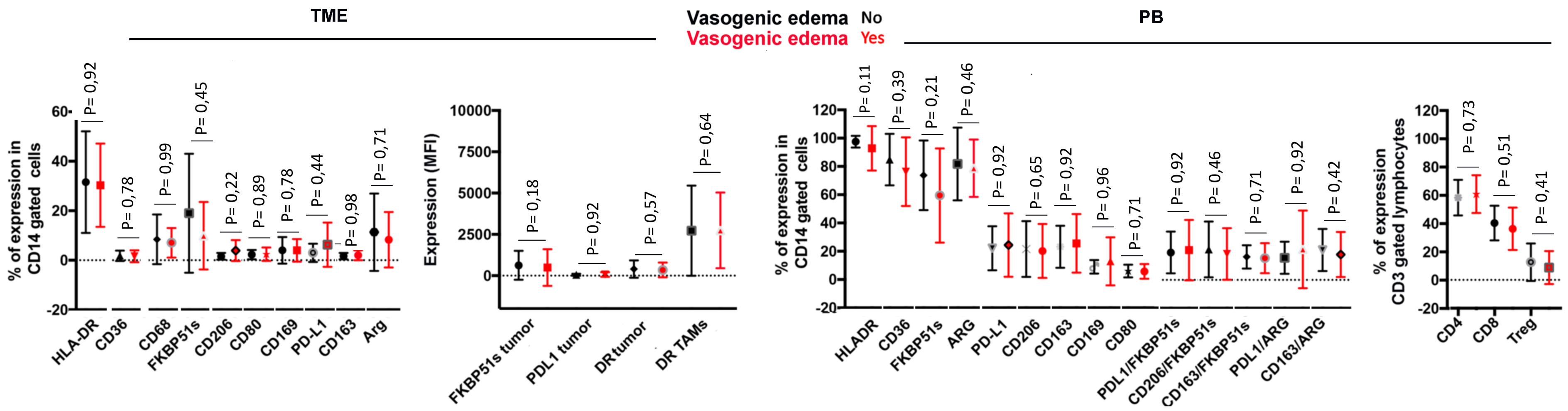

Fig S16. Vasogenic edema and Immunophenotype of TME and peripheral blood. Graphical representation of flow cytometry data of TME (graphs on the left) and peripheral blood (graphs on the right) from primary tumors. No Vasogenic edema, black histograms; Vasogenic edema, red histograms. P values are calculated using Mann Whitney test.
