# Supplementary material for: The FKBP51s Splice Isoform Predicts Unfavorable Prognosis in Patients with Glioblastoma
Source: Cancer Res Commun. 2024 May 16;4(5):1296–306. doi: 10.1158/2767-9764.CRC-24-0083 (PMC11097923; doi:10.1158/2767-9764.CRC-24-0083)
Supplement: Table S1 — Patients’ details [file crc-24-0083-s19.docx]

**Supplementary Table S1.** Patients’ details

| **Location** | **Age** | **Sex** | **P/R** | **EE*** | **CCI*** | **MS*** | **NS** | **ITSS** | **V** | **MH*** | **E*** | **ADC** | **HG*** | **OS** | **T-FK°** | **Cyt** |
| --- | --- | --- | --- | --- | --- | --- | --- | --- | --- | --- | --- | --- | --- | --- | --- | --- |
| **right front tempor** | 78 | M | **0** | **0** | **1** | **1** | **2** | **2** | **97,5** | **1** | **0** | **1.186** | **1** |  | **high** |  |
| **right front tempor** | 59 | F | **0** | **0** | **1** | **1** | **3** | **2** | **71** | **0** | **1** | **1108** | **1** |  | **high** |  |
| **left parietal** | 75 | F | **0** | **0** | **1** | **1** | **2** | **0** | **13,8** | **0** | **1** | **977** | **2** |  | **low** |  |
| **right temporal** | 62 | M | **0** | **0** | **1** | **0** | **2** | **1** | **32,8** | **0** | **1** | **660** | **1** |  | **low** |  |
| **right parietal** | 71 | F | **0** | **0** | **0** | **0** | **1** | **2** | **4,19** | **0** | **1** | **943** | **1** |  | **low** |  |
| **right pariet tempor** | 64 | M | **0** | **0** | **0** | **0** | **3** | **1** | **12,7** | **0** | **0** | **986** | **1** |  | **low** |  |
| **two right temporal** | 56 | M | **0** | **1** | **0** | **0** | **1** | **0** | **7,32** | **0** | **0** | **695** | **1** |  | **low** |  |
| **two right tempor frontal** | 65 | M | **0** | **0** | **0** | **1** | **2** | **ND** | **20,9** | **0** | **0** | **858** | **1** | **ND** | **low** |  |
| **left temporal frontal** | 56 | M | **0** | **0** | **0** | **1** | **3** | **2** | **50,8** | **0** | **0** | **873** | **2** |  | **high** |  |
| **right frontal** | 43 | F | **0** | **0** | **0** | **1** | **3** | **2** | **40,2** | **0** | **1** | **734** | **1** |  | **low** |  |
| **left frontal** | 74 | F | **0** | **0** | **0** | **0** | **3** | **2** | **7,42** | **0** | **0** | **460** | **PB** | **ND** | **high** |  |
| **left frontal** | 72 | M | **0** | **0** | **1** | **1** | **3** | **ND** | **68,6** | **ND** | **ND** | **990** | **2** |  | **high** |  |
| **left frontal temporal** | 55 | M | **0** | **0** | **1** | **1** | **3** | **2** | **68,1** | **1** | **1** | **750** | **2** |  | **high** |  |
| **left frontal** | 73 | M | **0** | **0** | **1** | **1** | **2** | **ND** | **33** | **0** | **1** | **1216** | **1** |  | **low** |  |
| **left pariet and periventricular** | 74 | F | **0** | **1** | **0** | **0** | **3** | **0** | **12,7** | **0** | **0** | **751** | **TME** |  | **high** | **ND** |
| **left frontal** | 60 | M | **0** | **0** | **1** | **0** | **3** | **1** | **8,01** | **0** | **1** | **1147** | **2** |  | **high** |  |
| **right frontal** | 43 | M | **0** | **0** | **0** | **0** | **3** | **2** | **12,5** | **0** | **0** | **1200** | **2** | **ND** | **high** |  |
| **right frontal parietal** | 75 | F | **0** | **0** | **1** | **1** | **2** | **0** | **26,1** | **0** | **1** | **720** | **2** |  | **high** |  |
| **left parietal** | 69 | F | **0** | **0** | **0** | **0** | **3** | **3** | **18,9** | **0** | **0** | **873** | **2** | **ND** | **high** |  |
| **left frontal temporal** | 49 | M | **0** | **0** | **0** | **1** | **1** | **2** | **180** | **0** | **1** | **994** | **2** |  | **high** |  |
| **left frontal temporal** | 69 | F | **0** | **0** | **1** | **1** | **3** | **3** | **20,6** | **0** | **1** | **1243** | **2** |  | **low** |  |
| **left temporal** | 60 | M | **0** | **0** | **0** | **0** | **0** | **0** | **ND** | **0** | **0** | **985** | **PB**** |  | **high** |  |
| **left frontal parietal** | 65 | M | **0** | **0** | **1** | **1** | **2** | **3** | **52,6** | **0** | **0** | **888** | **2** |  | **low** | **ND** |
| **left frontal** | 55 | M | **0** | **0** | **1** | **0** | **1** | **0** | **2,5** | **0** | **1** | **1177** | **2** |  | **low** | **ND** |
| **right frontal** | 60 | M | **0** | **0** | **1** | **1** | **1** | **1** | **6,2** | **0** | **1** | **1028** | **1** | **ND** | **low** | **ND** |
| **right frontal insular** | 55 | F | **0** | **0** | **0** | **1** | **3** | **3** | **73,4** | **0** | **0** | **754** | **1** |  | **low** | **ND** |
| **mutifocal, right hemisph** | 64 | M | **0** | **0** | **0** | **0** | **0** | **0** | **ND** | **0** | **0** | **816** | **2** |  | **high** |  |
| **left temporal** | 68 | M | **1** | **0** | **0** | **0** | **ND** | **ND** | **ND** | **ND** | **ND** | **1247** | **1** | **ND** | **low** |  |
| **left occipital** | 58 | M | **1** | **1** | **1** | **0** | **ND** | **ND** | **ND** | **ND** | **ND** | **695** | **1** |  | **low** |  |
| **left temporal** | 68 | M | **1** | **0** | **0** | **1** | **ND** | **ND** | **ND** | **ND** | **ND** | **1123** | **1** | **ND** | **low** |  |
| **right frontal** | 58 | F | **1** | **0** | **1** | **0** | **ND** | **ND** | **ND** | **ND** | **ND** | **1286** | **1** |  | **low** |  |
| **right temporal frontal** | 54 | M | **1** | **1** | **0** | **1** | **ND** | **ND** | **ND** | **ND** | **ND** | **541** | **1** |  | **high** |  |
| **right temporal** | 65 | M | **1** | **0** | **0** | **1** | **ND** | **ND** | **ND** | **ND** | **ND** | **1169** | **PB** | **ND** | **high** |  |
| **left temporal** | 64 | M | **1** | **0** | **0** | **0** | **ND** | **ND** | **ND** | **ND** | **ND** | **1345** | **2** | **ND** | **low** |  |
| **left parietal** | 63 | M | **1** | **1** | **1** | **0** | **ND** | **ND** | **ND** | **ND** | **ND** | **ND** | **1** |  | **high** |  |
| **right frontal** | 73 | F | **1** | **0** | **0** | **0** | **ND** | **ND** | **ND** | **ND** | **ND** | **1130** | **1** |  | **low** |  |
| **right frontal** | 61 | M | **1** | **1** | **1** | **0** | **ND** | **ND** | **ND** | **ND** | **ND** | **1218** | **2** |  | **high** | **ND** |

**Abbreviations:** P/R, Primary 0/Recurrence 1; EE, Ependymal enhancement; CCI, Corpus callosum infiltration; MS, Midline shift; NS, Necrosis score; ITSS, Intratumoral susceptibility signal score; V, Tumor volume (cm3); MH, Macroscopic hemorrage; E, Proportion of edema; ADC, apparent diffusion coefficient value 10-6 mm2/sec AR=20mm^2; HG, Heatmap group; OS, overall survival; T-FK, Tumor-FKBP51s; Cyt, Cytokine detection; PB peripheral blood but partial staining of TME (PDL-1, FKBP51s, HLADR); TME tumor microenvironment only, no peripheral blood available; ND, not available data.

* Yes 1, no 0; ** TME staining includes only PDL-1 and FKBP51s; ° Divided in high and low according to the median FKBP51s-tumor expression value.
