# Supplementary material for: The FKBP51s Splice Isoform Predicts Unfavorable Prognosis in Patients with Glioblastoma
Source: Cancer Res Commun. 2024 May 16;4(5):1296–306. doi: 10.1158/2767-9764.CRC-24-0083 (PMC11097923; doi:10.1158/2767-9764.CRC-24-0083)
Supplement: Table S2 — Tumor FKBP51-expression does not affect the composition of TME in CD163 or HLA- DR-TAMs nor does it influence tumor HLA-DR expression. Pearson r coefficient and p values are indicated for each variable. [file crc-24-0083-s20.docx]

**Supplementary Table S2** Tumor FKBP51-expression does not affect the composition of TME in CD163 or HLA-DR-TAMs nor does it influence tumor HLA-DR expression. Pearson r coefficient and p values are indicated for each variable.

| **TME-TAMs** | **Pearson r** | **p** |
| --- | --- | --- |
| CD163 | 0,19 | 0,27 |
| HLA-DR (%) | 0,17 | 0,3 |
| HLA-DR (MFI) | 0,18 | 0,28 |
| CD80 | 0,12 | 0,49 |

| **TUMOR** | **Pearson r** | **p** |
| --- | --- | --- |
| HLA-DR | 0,28 | 0,09 |
