# Supplementary material for: The FKBP51s Splice Isoform Predicts Unfavorable Prognosis in Patients with Glioblastoma
Source: Cancer Res Commun. 2024 May 16;4(5):1296–306. doi: 10.1158/2767-9764.CRC-24-0083 (PMC11097923; doi:10.1158/2767-9764.CRC-24-0083)
Supplement: Table S3 — Tumor FKBP51-expression does not affect the counts of the indicated peripheral blood TAM phenotypes nor does it influence CD4 and CD8 T lymphocytes’ counts. Pearson r coefficient and p values are indicated for each variable. [file crc-24-0083-s21.docx]

**Supplementary Table S3** Tumor FKBP51-expression does not affect the counts of the indicated peripheral blood TAM phenotypes nor does it influence CD4 and CD8 T lymphocytes’ counts. Pearson r coefficient and p values are indicated for each variable.

| **PB-TAMs and T cells** | | **Pearson r** | | **p** |
| --- | --- | --- | --- | --- |
| PDL-1/Arg | 0,19 | | 0,26 | |
| HLA-DR | 0,15 | | 0,3 | |
| Arg | 0,14 | | 0,24 | |
| CD36 | 0,089 | | 0,6 | |
| CD4 | 0,01 | | 0,91 | |
| CD8 | -0,004 | | 0,97 | |
| CD163/FKBP51s | -0,07 | | 0,6 | |
| CD163 | -0,22 | | 0,18 | |
| CD163/Arg | -0,29 | | 0,08 | |
