# Supplementary material for: The FKBP51s Splice Isoform Predicts Unfavorable Prognosis in Patients with Glioblastoma
Source: Cancer Res Commun. 2024 May 16;4(5):1296–306. doi: 10.1158/2767-9764.CRC-24-0083 (PMC11097923; doi:10.1158/2767-9764.CRC-24-0083)
Supplement: Table S4 — Tumor FKBP51s expression and MRI features. [file crc-24-0083-s22.docx]

**Supplementary Table S4** Tumor FKBP51s expression and MRI features

| **FKBP51s tumor MFI** | **vs.Prim recurr** | **vs. CCI** | **vs. MS** | **vs. NS** | **vs. TV(cm3)** | **vs. ADC** | **vs. VE** | **vs. EE** | **vs. MS** | **vs. ITSS** |
| --- | --- | --- | --- | --- | --- | --- | --- | --- | --- | --- |
| Pearson r | -0,12 | -0,03 | 0,07 | 0,30 | 0,13 | 0,00 | -0,07 | -0,04 | 0,07 | 0,10 |
| P (two-tailed) | 0,48 | 0,86 | 0,69 | 0,13 | 0,53 | 1,00 | 0,75 | 0,81 | 0,69 | 0,63 |
