# Supplementary material for: The FKBP51s Splice Isoform Predicts Unfavorable Prognosis in Patients with Glioblastoma
Source: Cancer Res Commun. 2024 May 16;4(5):1296–306. doi: 10.1158/2767-9764.CRC-24-0083 (PMC11097923; doi:10.1158/2767-9764.CRC-24-0083)
Supplement: Table S5 — Tumor volume and other MRI features: Pearson r coefficient and p values are indicated for each variable. Tumor volume is associated with midline shift. [file crc-24-0083-s23.docx]

**Supplementary Table S5** Tumor volume and other MRI features: Pearson r coefficient and p values are indicated for each variable. Tumor volume is associated with midline shift.

| Tumor volume (cm3) | vs. ADC value | vs. VE | vs. EE | vs. ITSS score | vs. MS | vs. NS |
| --- | --- | --- | --- | --- | --- | --- |
| Pearson r | 0,07998 | 0,07121 | -0,2089 | 0,38 | 0,5273 | -0,04779 |
| P (two-tailed) | 0,7039 | 0,7409 | 0,3163 | 0,0811 | 0,0081 | 0,8205 |
