# Supplementary material for: The FKBP51s Splice Isoform Predicts Unfavorable Prognosis in Patients with Glioblastoma
Source: Cancer Res Commun. 2024 May 16;4(5):1296–306. doi: 10.1158/2767-9764.CRC-24-0083 (PMC11097923; doi:10.1158/2767-9764.CRC-24-0083)
Supplement: Table S6 — Necrosis score and immunophenotyping values: Pearson r coefficient and p values are indicated for each variable. Linear correlation of necrosis score with tumor PDL-1 expression and PDL-1/FKBP51s PB-TAMs. [file crc-24-0083-s24.docx]

**Supplementary Table S6** Necrosis score and immunophenotyping values: Pearson r coefficient and p values are indicated for each variable. Linear correlation of necrosis score with tumor PDL-1 expression and PDL-1/FKBP51s PB-TAMs.

| Necrosis score vs TME-TAMs | | | | | | | | | |
| --- | --- | --- | --- | --- | --- | --- | --- | --- | --- |
|  | **FKBP51s tumor (MFI)** | **PDL1 tumor (MFI)** | **HLA-DR tumor (MFI)** | **HLA-DR-TAMs (MFI)** | **HLA-DR** | **CD36** | **CD68** |  |  |
| Pearson r | 0,34 | 0,39 | 0,14 | 0,05 | -0,07 | 0,39 | 0,17 |  |  |
| P (two-tailed) | 0,08 | **0,04** | 0,51 | 0,80 | 0,73 | 0,06 | 0,43 |  |  |
|  | **FKBP51s** | **CD206** | **CD80** | **CD169** | **PD-L1** | **CD163** | **Arg** |  |  |
| Pearson r | 0,33 | 0,20 | 0,02 | 0,17 | -0,04 | 0,39 | 0,38 |  |  |
| P (two-tailed) | 0,10 | 0,34 | 0,92 | 0,41 | 0,83 | **0,05** | 0,06 |  |  |
|  |  | | | | | | | | |
| Necrosis score vs PB-TAMs | | | | | | | | | |
|  | **HLA-DR** | **CD36** | **FKBP51s** | **ARG** | **PD-L1** | **CD206** | **CD163** | **CD169** |  |
| Pearson r | 0,26 | -0,33 | 0,32 | -0,11 | 0,22 | 0,24 | 0,08 | 0,19 |  |
| P (two-tailed) | 0,21 | 0,10 | 0,12 | 0,60 | 0,28 | 0,24 | 0,69 | 0,36 |  |
|  | **CD80** | **PDL1/**  **FKBP51s** | **CD206/**  **FKBP51s** | **CD163/**  **FKBP51s** | **PDL1/ARG** | **CD163/ARG** | **CD4** | **CD8** | **Treg** |
| Pearson r | -0,24 | 0,38 | 0,27 | 0,28 | -0,20 | 0,16 | -0,01 | -0,03 | 0,26 |
| P (two-tailed) | 0,24 | **0,05** | 0,18 | 0,16 | 0,33 | 0,44 | 0,96 | 0,90 | 0,20 |
