# Supplementary material for: The FKBP51s Splice Isoform Predicts Unfavorable Prognosis in Patients with Glioblastoma
Source: Cancer Res Commun. 2024 May 16;4(5):1296–306. doi: 10.1158/2767-9764.CRC-24-0083 (PMC11097923; doi:10.1158/2767-9764.CRC-24-0083)
Supplement: Table S7 — Necrosis score in relation to other MRI features: Pearson r coefficient and p values are indicated for each variable. Correlation between necrosis score and ITSS. [file crc-24-0083-s25.docx]

**Supplementary Table S7** Necrosis score in relation to other MRI features: Pearson r coefficient and p values are indicated for each variable. Correlation between necrosis score and ITSS.

| Necrosis score | vs. TV | vs. ADC value | vs. VE | vs. EE | vs. ITSS score | vs. MS | vs. CCI |
| --- | --- | --- | --- | --- | --- | --- | --- |
| Pearson r | -0,04779 | -0,05367 | -0,0395 | -0,0432 | 0,5252 | 0,2134 | 0,08207 |
| P (two-tailed) | 0,8205 | 0,7903 | 0,8481 | 0,8306 | 0,0084 | 0,2851 | 0,684 |
