# Supplementary material for: The FKBP51s Splice Isoform Predicts Unfavorable Prognosis in Patients with Glioblastoma
Source: Cancer Res Commun. 2024 May 16;4(5):1296–306. doi: 10.1158/2767-9764.CRC-24-0083 (PMC11097923; doi:10.1158/2767-9764.CRC-24-0083)
Supplement: Table S8 — ITSS score and immunophenotyping values: Pearson r coefficient and p values are indicated for each variable. [file crc-24-0083-s26.docx]

**Supplementary Table S8** ITSS score and immunophenotyping values: Pearson r coefficient and p values are indicated for each variable.

| ITSS vs TME-TAMs | | | | | | | | | |
| --- | --- | --- | --- | --- | --- | --- | --- | --- | --- |
|  | **FKBP51s tumor (MFI)** | **PDL1 tumor (MFI)** | **HLA-DR tumor (MFI)** | **HLA-DR TAMs (MFI)** | **HLADR** | **CD36** | **CD68** |  |  |
| Pearson r | 0,07 | 0,12 | 0,29 | 0,12 | 0,17 | -0,13 | -0,20 |  |  |
| P (two-tailed) | 0,75 | 0,58 | 0,18 | 0,57 | 0,45 | 0,58 | 0,38 |  |  |
|  | **FKBP51s** | **CD206** | **CD80** | **CD169** | **PD-L1** | **CD163** | **Arg** |  |  |
| Pearson r | -0,03 | -0,07 | -0,14 | -0,21 | 0,08 | 0,04 | 0,09 |  |  |
| P (two-tailed) | 0,88 | 0,74 | 0,53 | 0,34 | 0,73 | 0,87 | 0,69 |  |  |
|  |  |  |  |  |  |  |  |  |  |
| ITSS vs PB-TAMs | | | | | | | | | |
|  | **HLA-DR** | **CD36** | **FKBP51s** | **ARG** | **PD-L1** | **CD206** | **CD163** | **CD169** |  |
| Pearson r | 0,20 | -0,06 | 0,17 | -0,25 | -0,11 | 0,13 | -0,21 | 0,10 |  |
| P (two-tailed) | 0,37 | 0,77 | 0,44 | 0,25 | 0,61 | 0,55 | 0,33 | 0,65 |  |
|  | **CD80** | **PDL1/**  **FKBP51s** | **CD206/**  **FKBP51s** | **CD163/**  **FKBP51s** | **PDL1/ARG** | **CD163/ARG** | **CD4** | **CD8** | **Treg** |
| Pearson r | -0,12 | 0,03 | 0,15 | -0,10 | -0,29 | -0,10 | 0,13 | -0,16 | 0,18 |
| P (two-tailed) | 0,58 | 0,90 | 0,49 | 0,65 | 0,18 | 0,64 | 0,56 | 0,47 | 0,42 |
